# Supplementary material for: SymProFold: Structural prediction of symmetrical biological assemblies
Source: Nat Commun. 2024 Sep 18;15:8152. doi: 10.1038/s41467-024-52138-3 (PMC11410804; doi:10.1038/s41467-024-52138-3)
Supplement: Supplementary file 1 — Supplementary Information [file 41467_2024_52138_MOESM1_ESM.pdf]

Supplementary Information to:

SymProFold: Structural prediction of symmetrical biological assemblies

Author list

Christoph Buhlheller[1,2#], Theo Sagmeister[1#], Christoph Grininger[1], Nina Gubensäk[1], Uwe B. Sleytr[3], Isabel Usón[4,5], Tea Pavkov-Keller[1,6,7*]

Affiliations

[1] Institute of Molecular Biosciences, University of Graz, 8010 Graz, Austria

[2] Medical University of Graz, 8010 Graz, Austria.

[3] Institute of Nanobiotechnology, University of Natural Resources and Life Sciences Vienna, 1190 Vienna, Austria

[4] Structural Biology Unit, Institute of Molecular Biology of Barcelona, Spanish National Research Council, 08028 Barcelona, Spain

[5] ICREA, Institució Catalana de Recerca i Estudis Avançats, 08003 Barcelona, Spain

[6] Field of Excellence BioHealth, University of Graz, 8010 Graz, Austria

[7] BioTechMed-Graz, University of Graz, 8010 Graz, Austria

[#] These authors contributed equally; names are in alphabetical order

* To whom correspondence should be addressed. Tel: +43 3163805483; e-mail: tea.pavkov@uni-graz.at

Supplementary Information content:

- Supplementary Figures 1-38
- Supplementary Tables 1-7
- Supplementary Methods 1-8
- Supplementary References 1-36

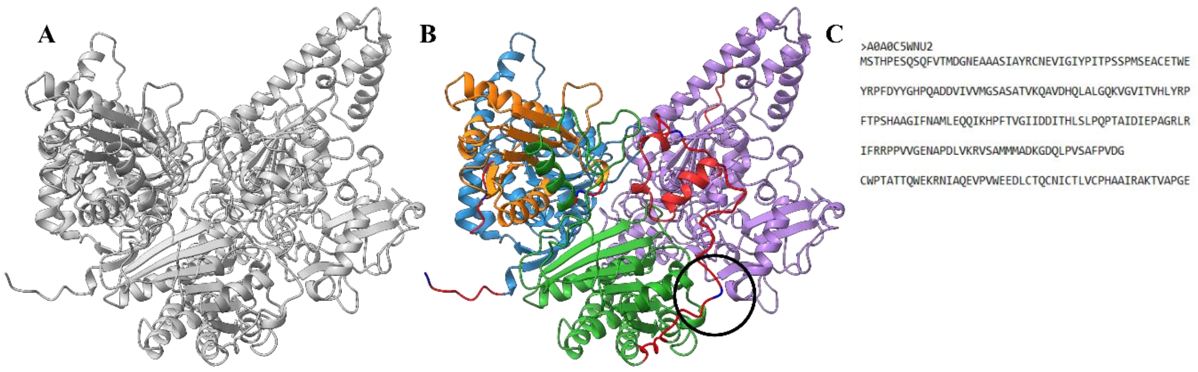

Supplementary Figure 1: Output of Domain_Separator of pyruvate-flavodoxin oxidoreductase from *Photobacterium gaetbulicola* Domain_Separator uses a predicted structure **A**) and creates a ChimeraX [1] session with the found domains colored separately. **B**) Domain_separator identified domains of A0A0C5WNU2 are colored in light blue, orange, purple, and green. Linker regions are colored red, and the amino acid, where the sequence gets cut, is colored dark blue (circled in B). **C)** shows the output of Domain_Separator, a fasta file with the sequence separated by line breaks. Each line equals one domain. (UniProt A0A0C5WNU2 [https://www.uniprot.org/uniprotkb/A0A0C5WNU2/entry]**.**

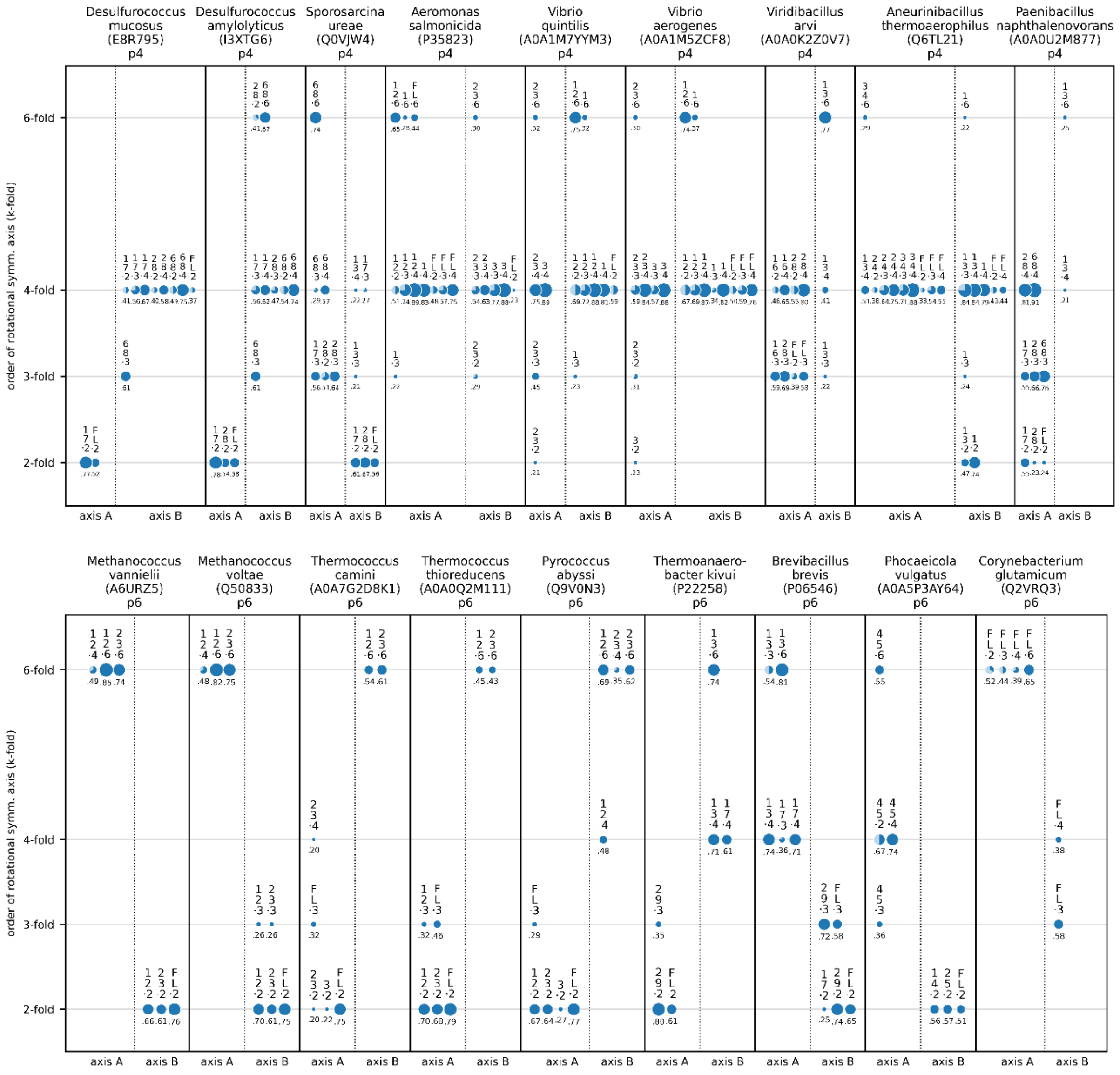

Supplementary Figure 2. Clustering of predicted symmetry complexes for different S-layer species. Comparison of symmetry axis orders (fold) determined by ab initio prediction. The circle diameters represent the respective ranking score. In this Figure, for each species, two clusters with non-coinciding symmetry axes and the highest ipTM+pTM scores are shown. For each subchain, the symmetry complex with the highest ipTM+pTM score is shown.

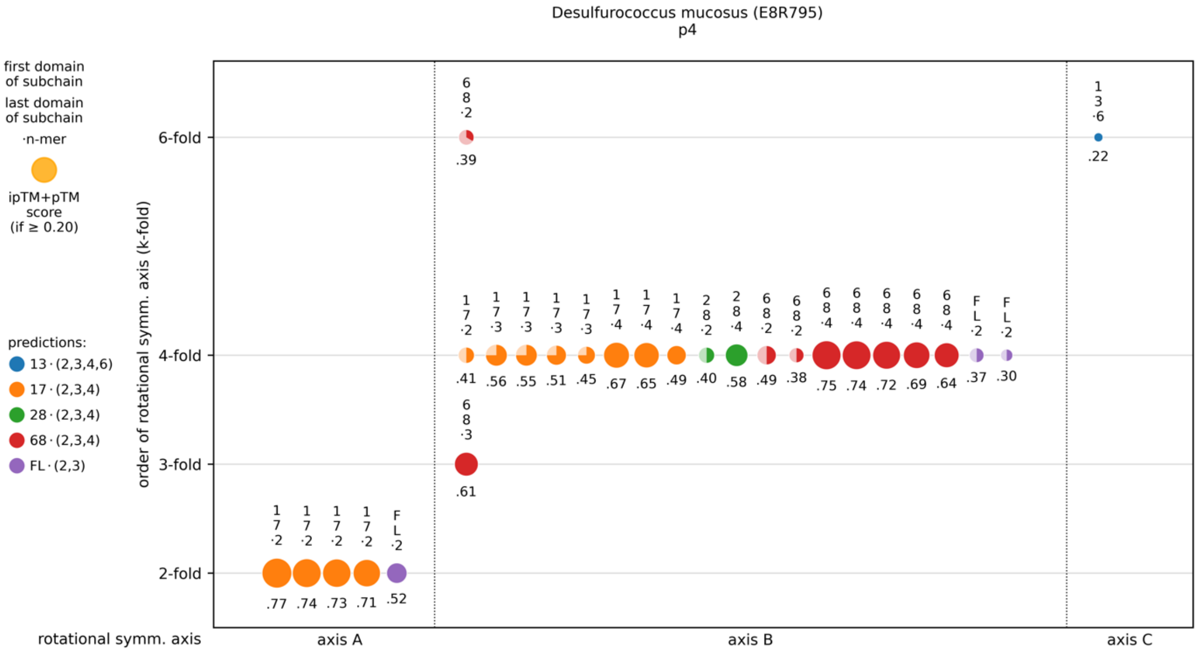

Supplementary Figure 3. Clustering of predicted symmetry complexes for *Desulfurococcus mucosus* S-layer (E8R795). The circle diameters represent the respective ranking scores, each color represents a subchain. For axis A cluster, prediction scores clearly indicate a 2-fold symmetry, for axis B a 4-fold symmetry axis. The 4-fold symmetry for cluster B is also supported insofar as the same subchain leads to the same order of symmetry (k-fold) for different molecule counts (n-mer) in the prediction. Analysis is based on X75 predictions.

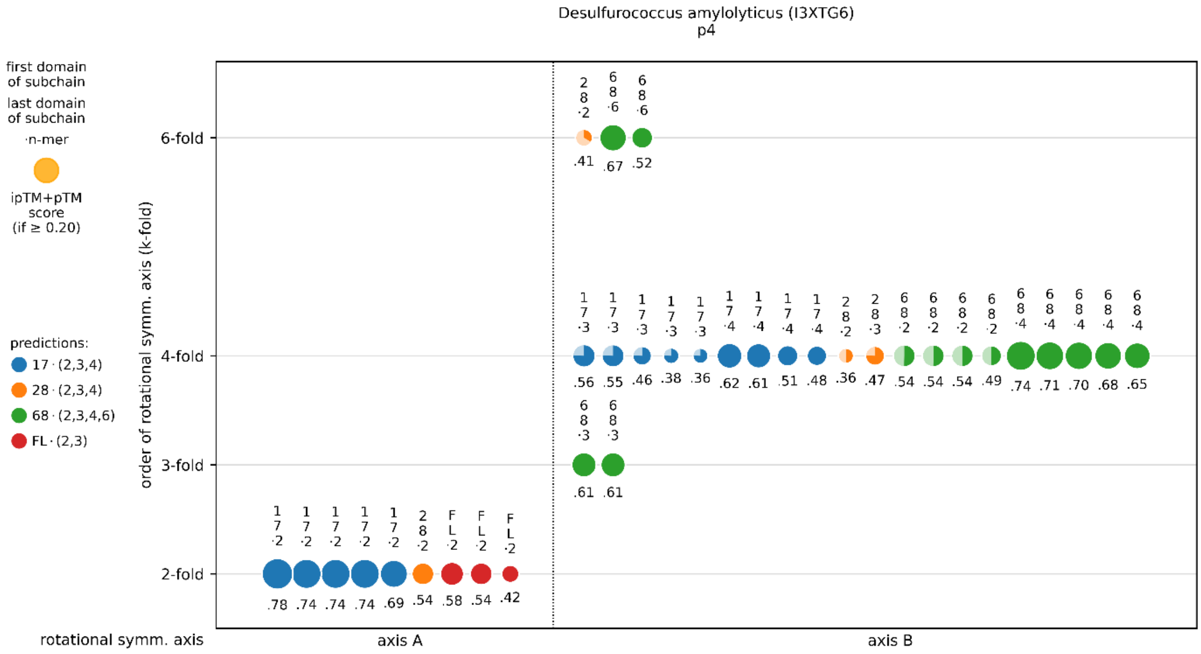

Supplementary Figure 4. Clustering of predicted symmetry complexes for *Desulfurococcus amylolyticus* S-layer (I3XTG6). The circle diameters represent the respective ipTM+pTM scores, each color represents a subchain. For axis A cluster, prediction scores clearly indicate a 2-fold symmetry, for axis B a 4-fold symmetry axis. The 4-fold symmetry for cluster B is also supported insofar as the same subchain leads to the same order of symmetry (k-fold) for different molecule counts (n-mer) in the prediction. Analysis is based on 60 predictions.

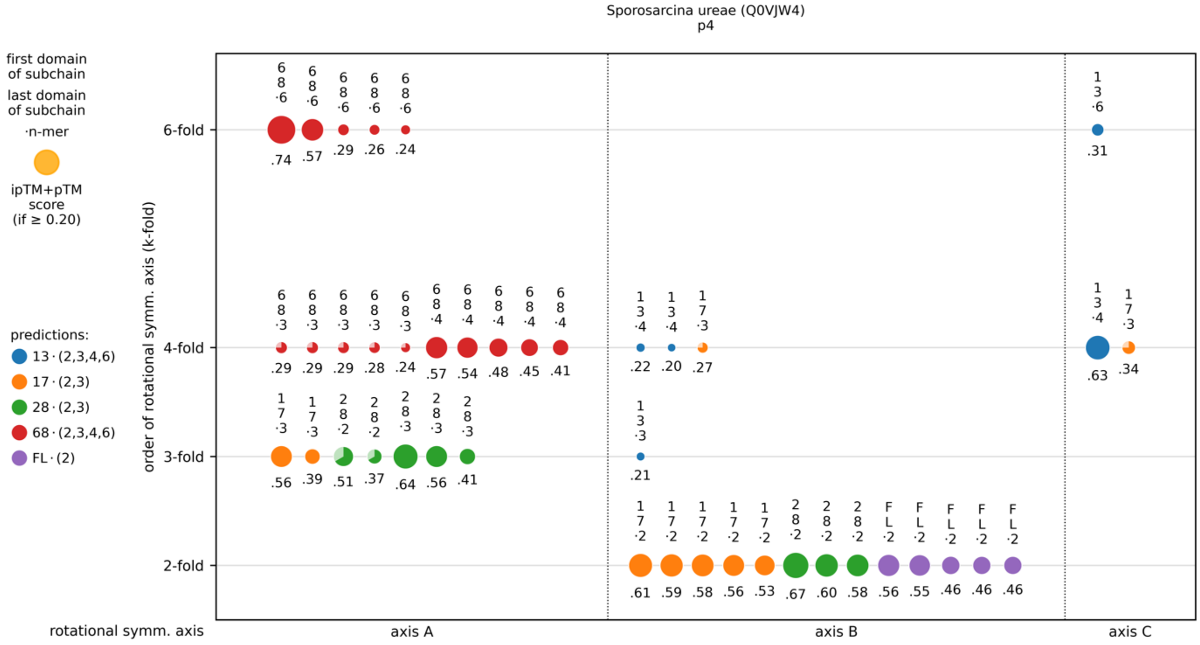

Supplementary Figure 5. Clustering of predicted symmetry complexes for *Sporosarcina ureae* S-layer (Q0VJW4). The circle diameters represent the respective ipTM+pTM scores, each color represents a subchain. For axis B cluster, prediction scores indicate a 2-fold symmetry. For axis A cluster, prediction scores indicate a 6-fold symmetry axis as first, then a 3-fold and a 4-fold symmetry. A 4-fold symmetry for cluster A is supported insofar as the same subchain leads to the same order of symmetry (4-fold) for different molecule counts (3-mer, 4-mer) in the prediction. A 6-fold symmetry for cluster A is not supported by different molecule counts in the prediction. Layer assembly without large number of clashes is possible with a 4-fold symmetry for cluster A, not for 6-fold symmetry. Analysis is based on 65 predictions.

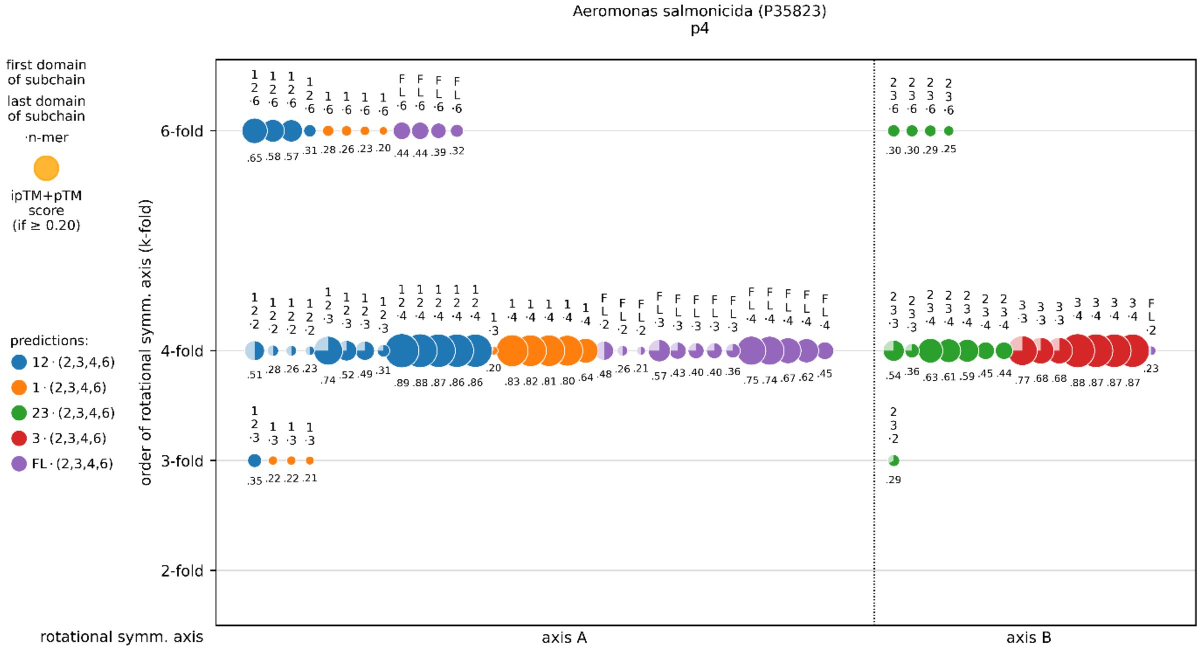

Supplementary Figure 6. Clustering of predicted symmetry complexes for *Aeromonas salmonicida* S-layer (P35823). The circle diameters represent the respective ipTM+pTM scores, each color represents a subchain. For axis A cluster and axis B cluster, prediction scores clearly indicate a 4-fold symmetry axis each. Both 4-fold symmetries are also supported insofar as the same subchains lead to the same order of symmetry (4-fold) for different molecule counts (n-mer) in the prediction. Analysis is based on 100 predictions.

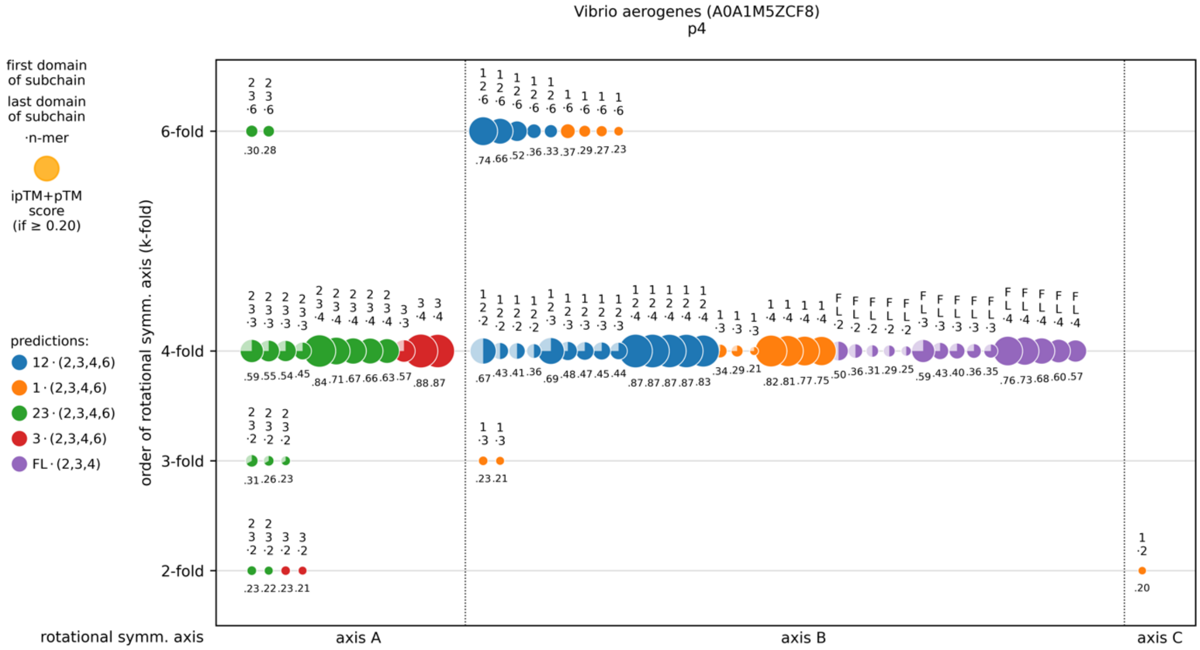

Supplementary Figure 7. Clustering of predicted symmetry complexes for *Vibrio aerogenes* S-layer (A0A1M5ZCF8). The circle diameters represent the respective ipTM+pTM scores, each color represents a subchain. For axis A cluster and axis B cluster, prediction scores clearly indicate a 4-fold symmetry axis each. Both 4-fold symmetries are also supported insofar as the same subchains lead to the same order of symmetry (4-fold) for different molecule counts (n-mer) in the prediction. Analysis is based on 95 predictions.

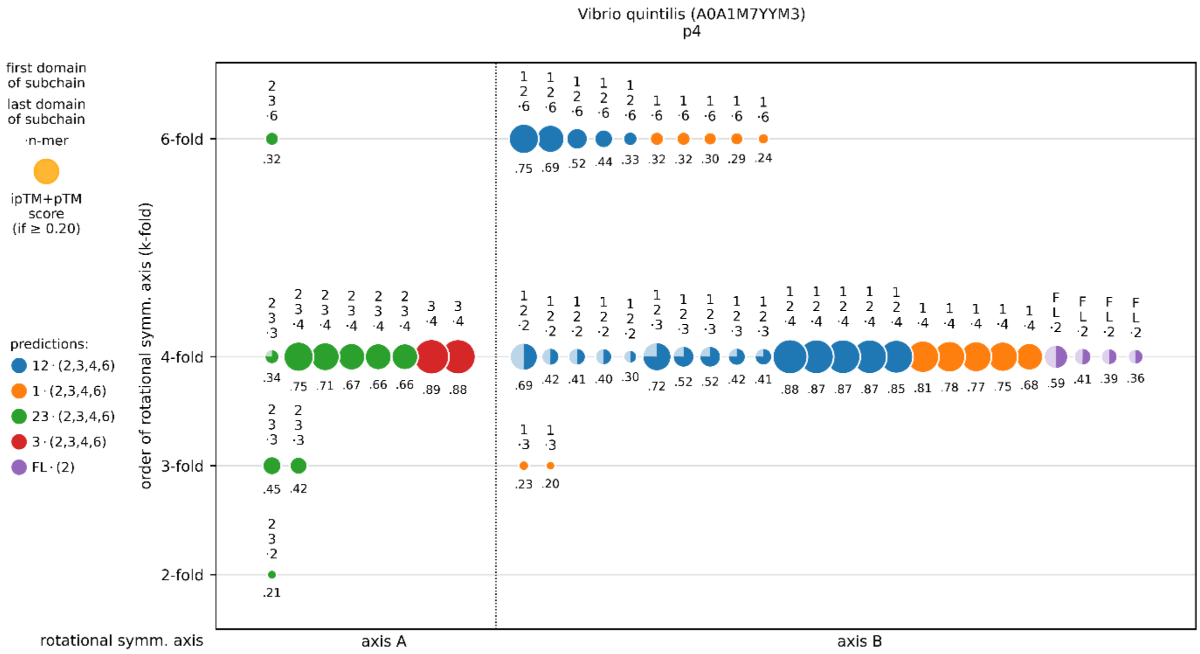

Supplementary Figure 8. Clustering of predicted symmetry complexes for Vibrio quintilis (A0A1M7YYM3). The circle diameters represent the respective ranking scores, each color represents a subchain. For axis A cluster and axis B clusters, prediction scores clearly indicate a 4-fold symmetry axis each. Both 4-fold symmetries are also supported insofar as the same subchains lead to the same order of symmetry (4-fold) for different molecule counts (n-mer) in the prediction. Analysis is based on 85 predictions.

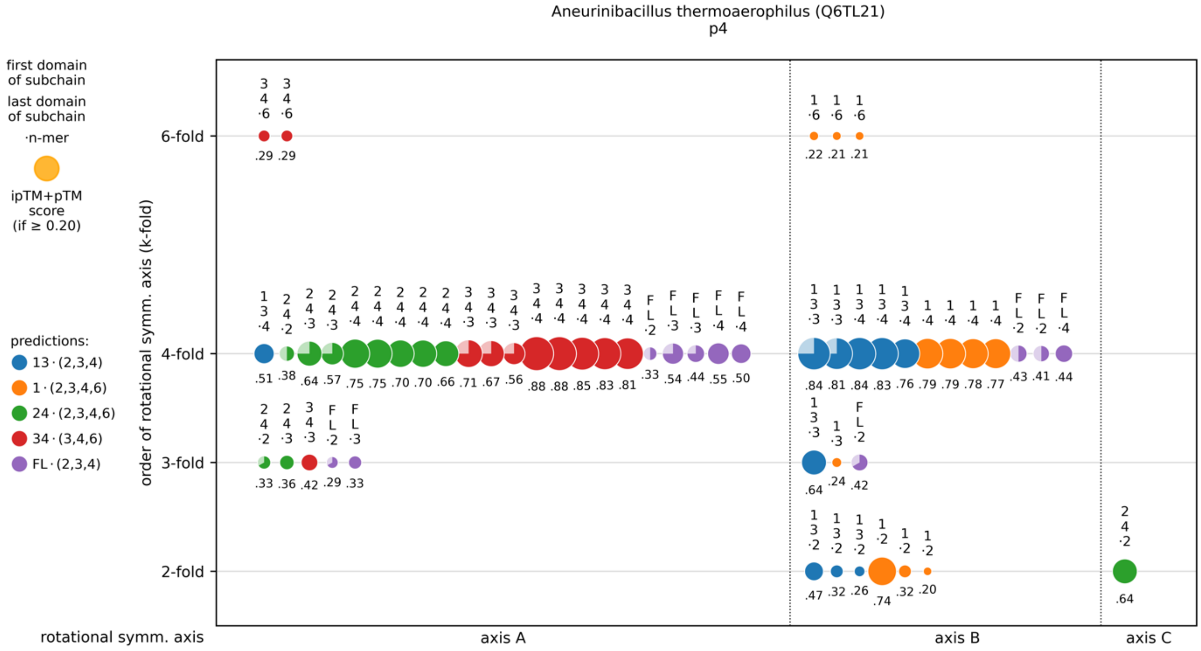

Supplementary Figure 9. Clustering of predicted symmetry complexes for *Aneurinibacillus thermoaerophilus* S-layer (Q6TL21). The circle diameters represent the respective ipTM+pTM scores, each color represents a subchain. Axis A cluster and axis B cluster show the highest prediction scores, clearly indicating a 4-fold axis each. Both 4-fold symmetries are also supported insofar as the same subchains lead to the same order of symmetry (4-fold) for different molecule counts (n-mer) in the prediction. Analysis is based on 85 predictions.

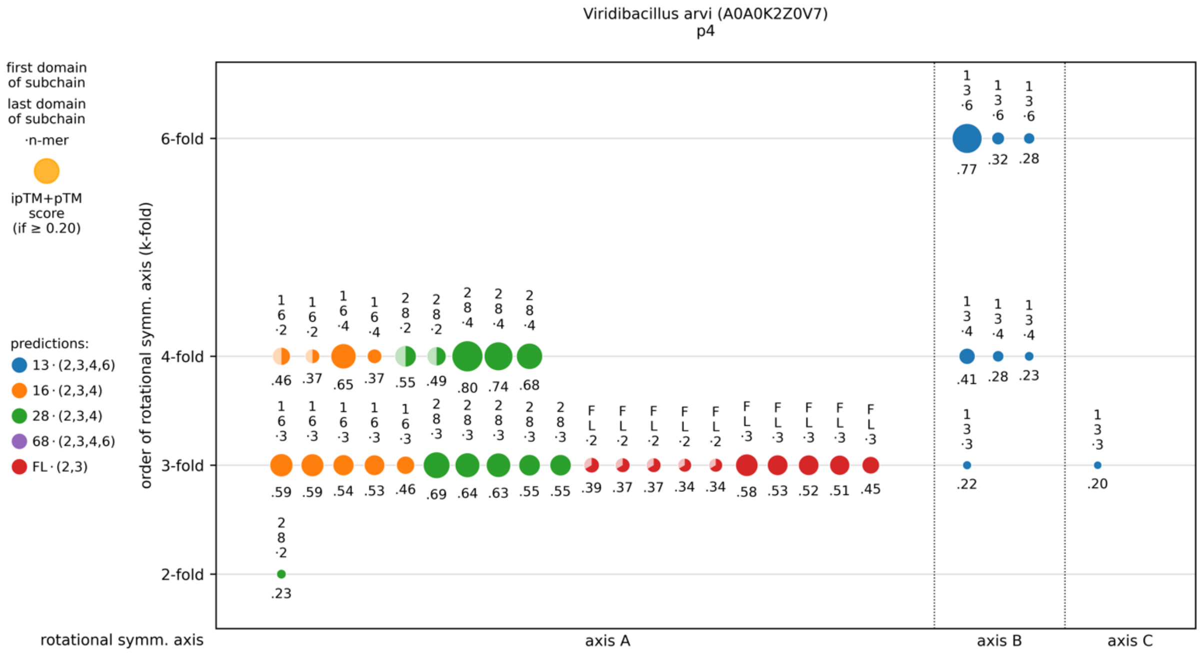

Supplementary Figure 10. Clustering of predicted symmetry complexes for *Viridibacillus arvi* S-layer (slp1, A0A0K2Z0V7). The circle diameters represent the respective ipTM+pTM scores, each color represents a subchain. For axis A cluster, prediction scores indicate a 4-fold symmetry axis as first and a 3-fold symmetry as second. For axis B cluster, prediction scores indicate a 6-fold symmetry as first and a 4-fold symmetry as second. Analysis is based on 80 predictions.

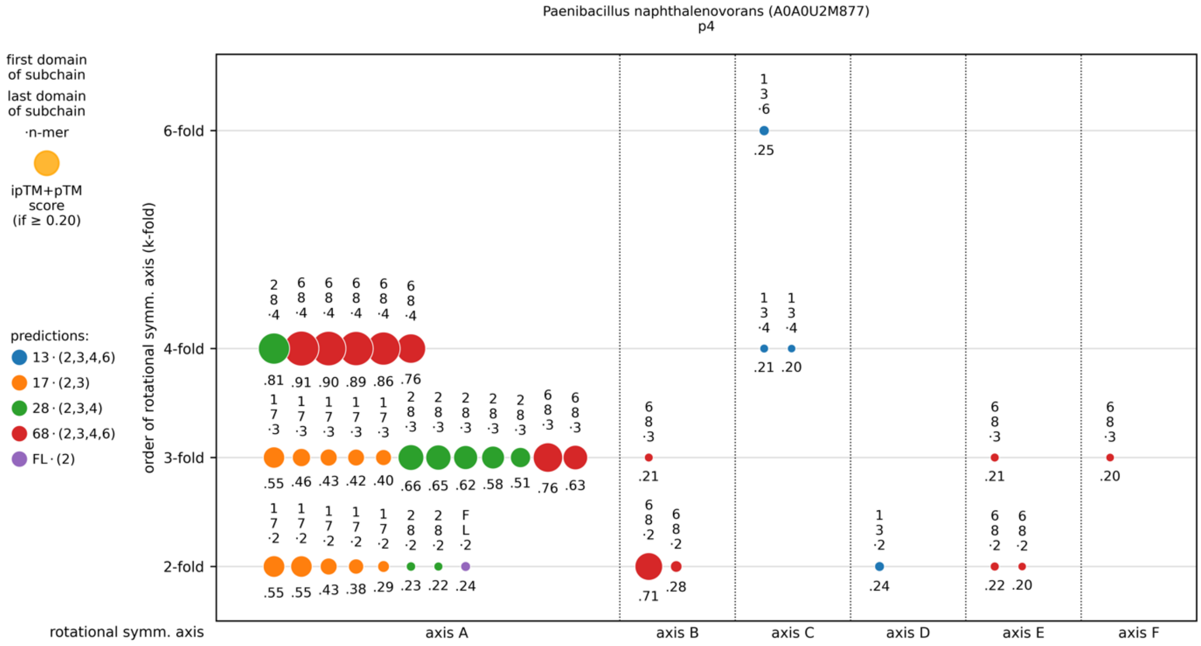

Supplementary Figure 11. Clustering of predicted symmetry complexes for *Paenibacillus naphthalenovorans* S-layer (A0A0U2M877). The circle diameters represent the respective ipTM+pTM scores, each color represents a subchain. Axes A and B coincide, prediction scores indicate a 4-fold symmetry axis. Layer assembly is possible with a 4-fold symmetry for cluster C. Analysis is based on 70 predictions.

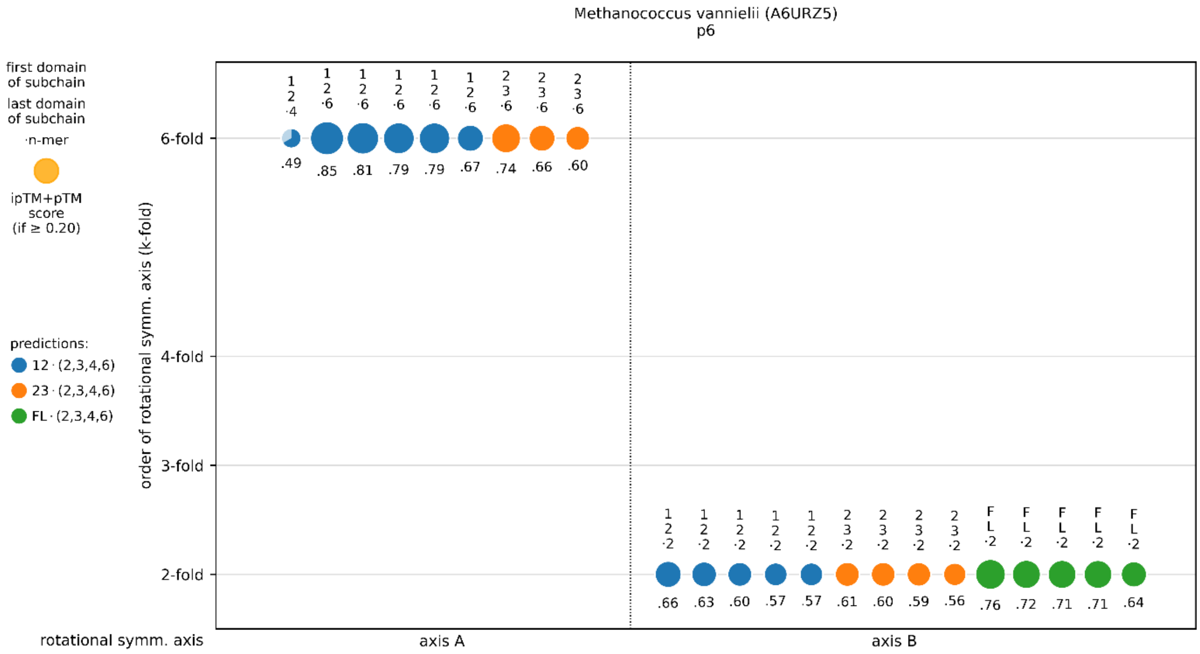

Supplementary Figure 12. Clustering of predicted symmetry complexes for *Methanococcus vannielii* S-layer (A6URZ5). The circle diameters represent the respective ipTM+pTM scores, each color represents a subchain. For axis A cluster, prediction scores clearly indicate a 6-fold symmetry, for axis B clearly a 2-fold symmetry axis. The 6-fold symmetry for cluster A is also supported insofar as the same subchain leads to the same order of symmetry (6-fold) for different molecule counts (4-mer, 6-mer) in the prediction. Analysis is based on 60 predictions.

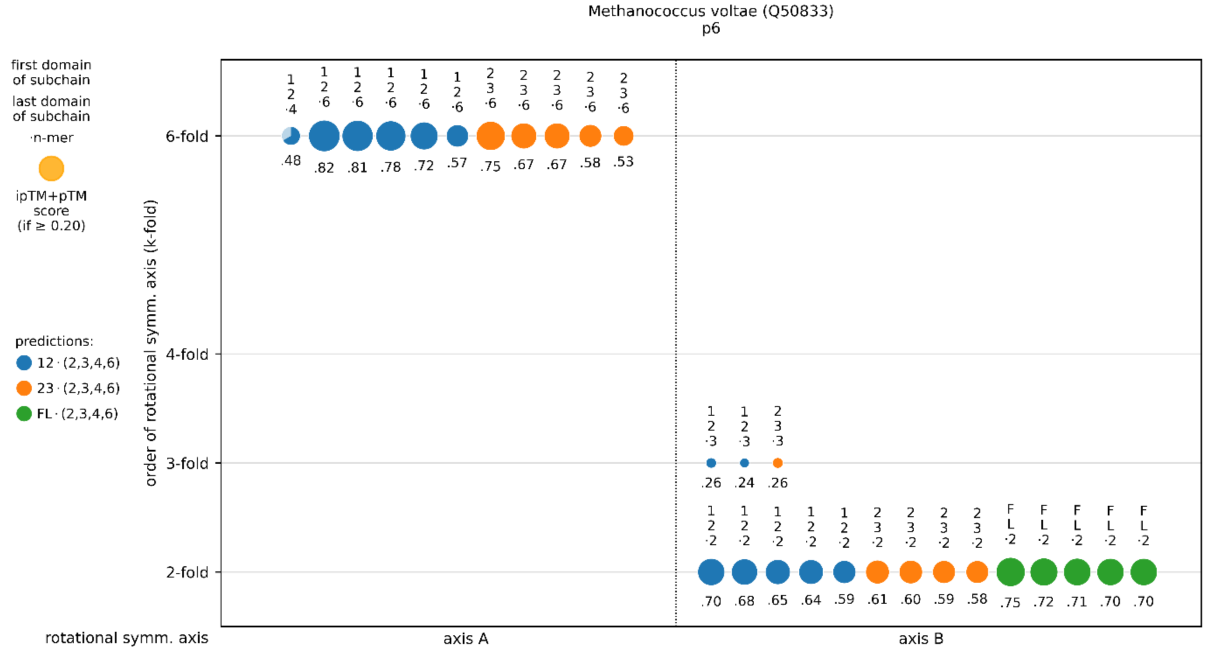

Supplementary Figure 13. Clustering of predicted symmetry complexes for *Methanococcus voltae* S-layer (Q50833). The circle diameters represent the respective ipTM+pTM scores, each color represents a subchain. For axis A cluster, prediction scores clearly indicate a 6-fold symmetry, for axis B clearly a 2-fold symmetry axis. The 6-fold symmetry for cluster A is also supported insofar as the same subchain leads to the same order of symmetry (6-fold) for different molecule counts (4-mer, 6-mer) in the prediction. Analysis is based on 60 predictions.

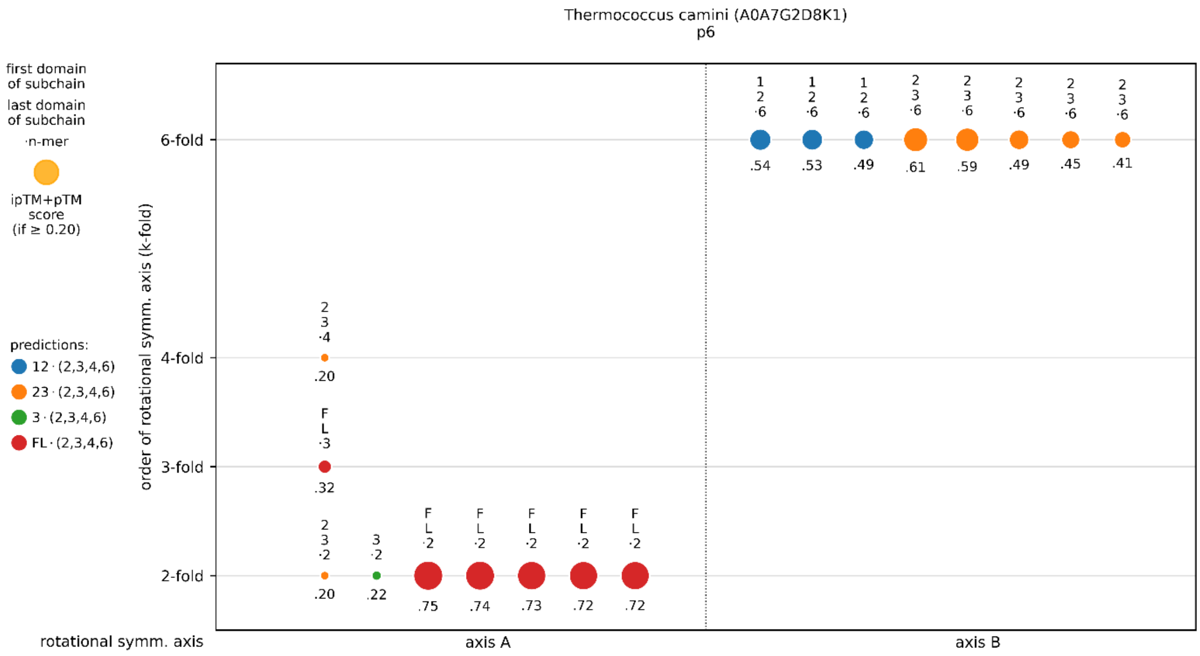

Supplementary Figure 14. Clustering of predicted symmetry complexes for *Thermococcus camini* S-layer (A0A7G2D8K1). The circle diameters represent the respective ipTM+pTM scores, each color represents a subchain. For axis A cluster, prediction scores clearly indicate a 2-fold symmetry, for axis B clearly a 6-fold symmetry axis. Analysis is based on 80 predictions.

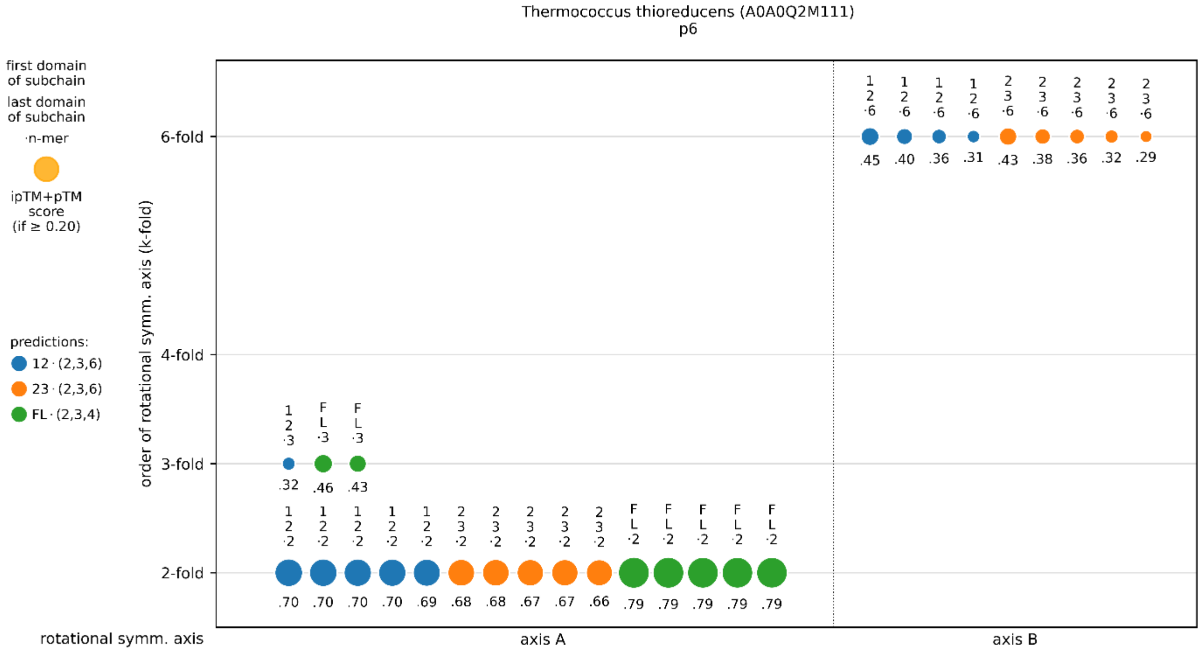

Supplementary Figure 15. Clustering of predicted symmetry complexes for *Thermococcus thioreducens* S-layer (A0A0Q2M111). The circle diameters represent the respective ipTM+pTM scores, each color represents a subchain. For axis A cluster, prediction scores indicate a 2-fold symmetry axis. For axis B cluster, prediction scores clearly indicate a 6-fold symmetry. Analysis is based on 45 predictions.

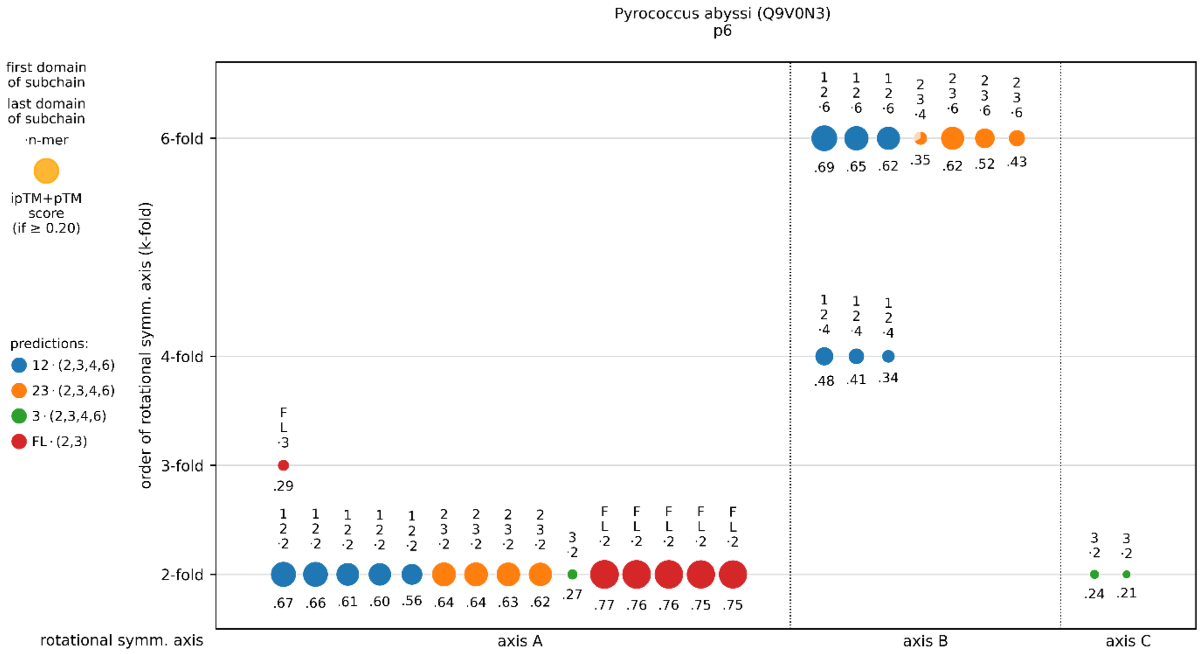

Supplementary Figure 16. Clustering of predicted symmetry complexes for *Pyrococcus abyssi* S-layer (Q9V0N3). The circle diameters represent the respective ipTM+pTM scores, each color represents a subchain. For axis A cluster, prediction scores clearly indicate a 2-fold symmetry. For axis B cluster, prediction scores clearly indicate a 6-fold symmetry. Analysis is based on 70 predictions.

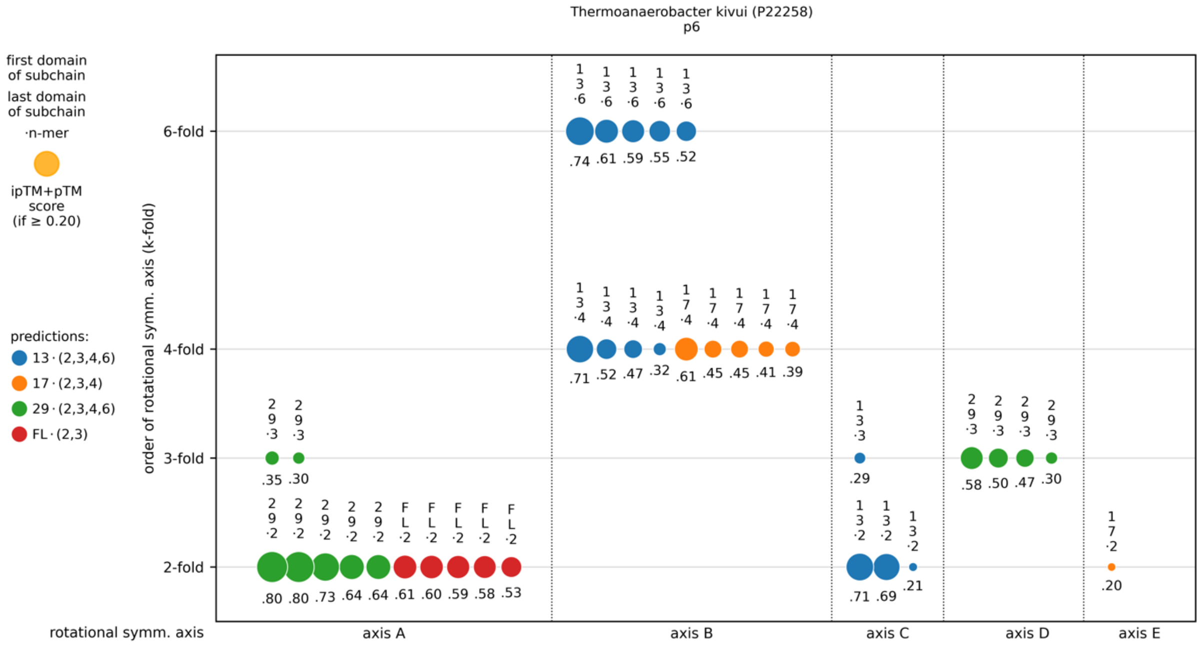

Supplementary Figure 17. Clustering of predicted symmetry complexes for *Thermoanaerobacter kivui* S-layer (P22258). The circle diameters represent the respective ipTM+pTM scores, each color represents a subchain. For axis A cluster, prediction scores clearly indicate a 2-fold symmetry axis. Axes B and C coincide, prediction scores indicate a 6-fold symmetry. Analysis is based on 65 predictions.

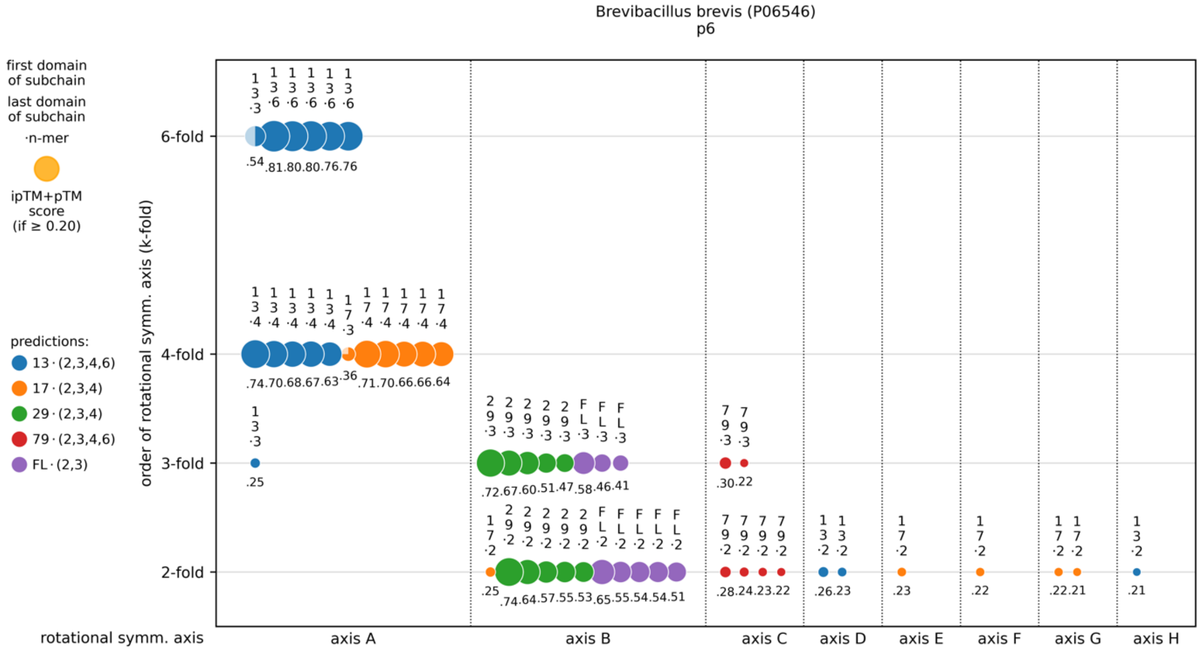

Supplementary Figure 18. Clustering of predicted symmetry complexes for *Brevibacillus brevis* S-layer (P06546). The circle diameters represent the respective ipTM+pTM scores, each color represents a subchain. For axis A cluster, prediction scores indicate a 6-fold symmetry. For axis B cluster, prediction scores indicate a 2-fold symmetry. Analysis is based on 80 predictions.

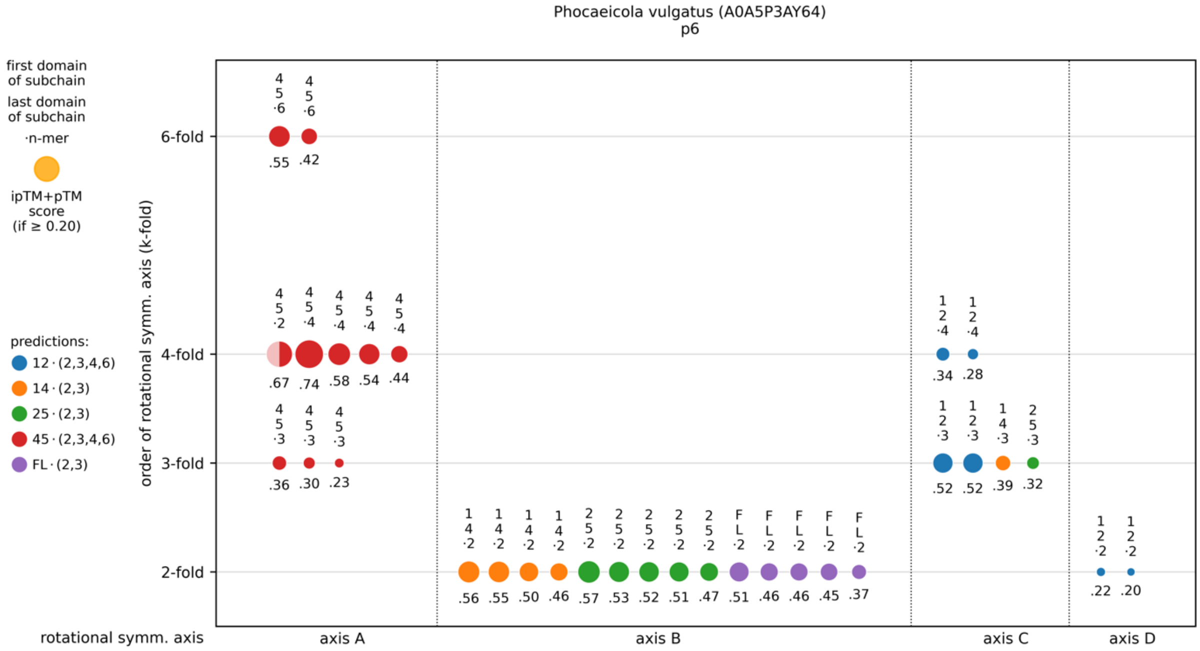

Supplementary Figure 19. Clustering of predicted symmetry complexes for *Phocaeicola vulgatus* S-layer (A0A5P3AY64). The circle diameters represent the respective ipTM+pTM scores, each color represents a subchain. For axis B cluster, prediction scores indicate a 2-fold symmetry. For axis A cluster, prediction scores indicate a 4-fold symmetry axis as first and a 6-fold symmetry as second. For axis C cluster, prediction scores indicate a 3-fold symmetry. Layer assembly is possible with a 6-fold symmetry for cluster A. Cluster B contains the 2-fold axis of rotational symmetry, cluster C the 3-fold axis of rotational symmetry in the resulting p6 lattice. Analysis is based on 70 predictions.

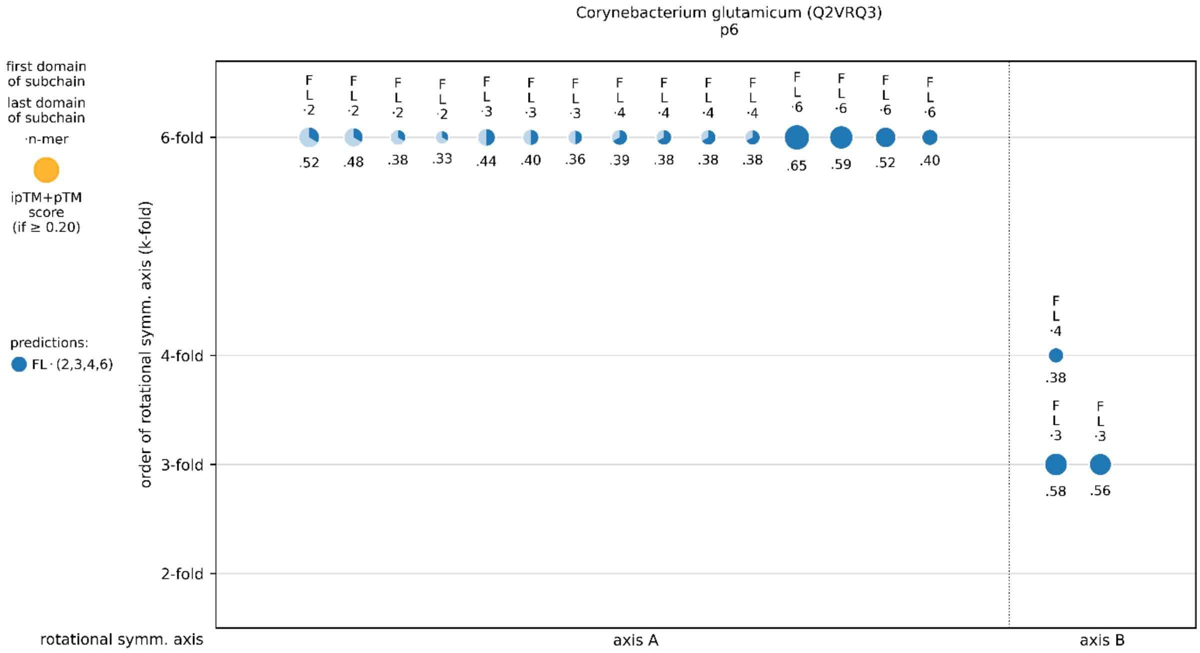

Supplementary Figure 20. Clustering of predicted symmetry complexes for *Corynebacterium glutamicum* S-layer (Q2VRQ3). The circle diameters represent the respective ipTM+pTM scores, each color represents a subchain. For axis A, prediction scores indicate a 6-fold symmetry axis. The 6-fold symmetry for cluster A is also supported insofar as the same subchain leads to the same order of symmetry (6-fold) for different molecule counts (2-mer, 3-mer, 4-mer, 6-mer) in the prediction. For axis B, prediction scores indicate a 3-fold symmetry axis. Analysis is based on 20 predictions.

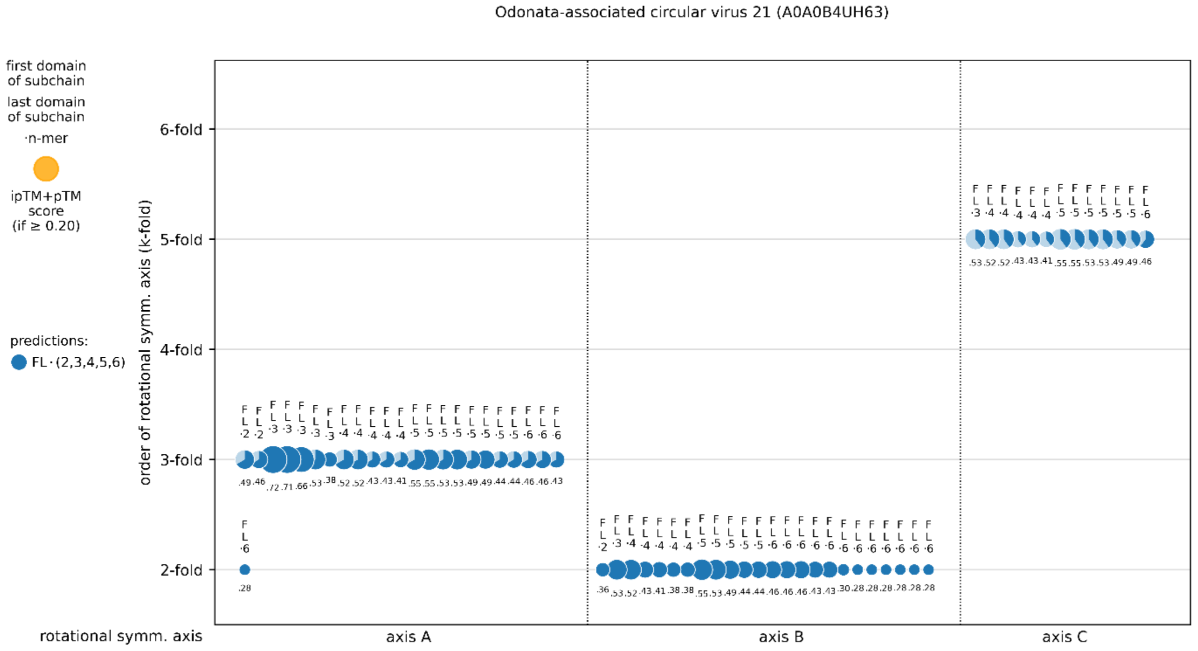

Supplementary Figure 21. Clustering of predicted symmetry complexes for the viral capsid of *Odonata-associated circular virus 21* (A0A0B4UH63). The circle diameters represent the respective ipTM+pTM scores. A prediction can contain more than one rotational symmetry axis. In these cases, there are several data points per prediction in the diagram. For axis A cluster, prediction scores clearly indicate a 3-fold symmetry, for axis B cluster a 2-fold symmetry and for axis C cluster a 5-fold symmetry. Analysis is based on 25 predictions.

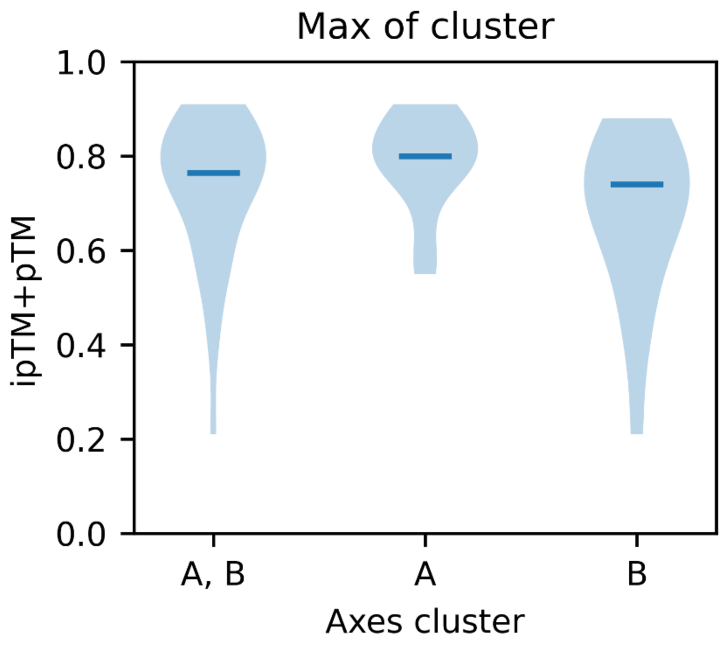

Supplementary Figure 22. ipTM+pTM scores of the predicted S-layer models published in this study. Data basis are complete symmetry complexes (models) that show the predicted order of symmetry. For each axis cluster, the highest-ranking model is included in the statistics. Violin plot, from left to right: axes A+B taken together, only axis A, only axis B. Dark blue line represents the median. Source data are provided as a Source Data file.

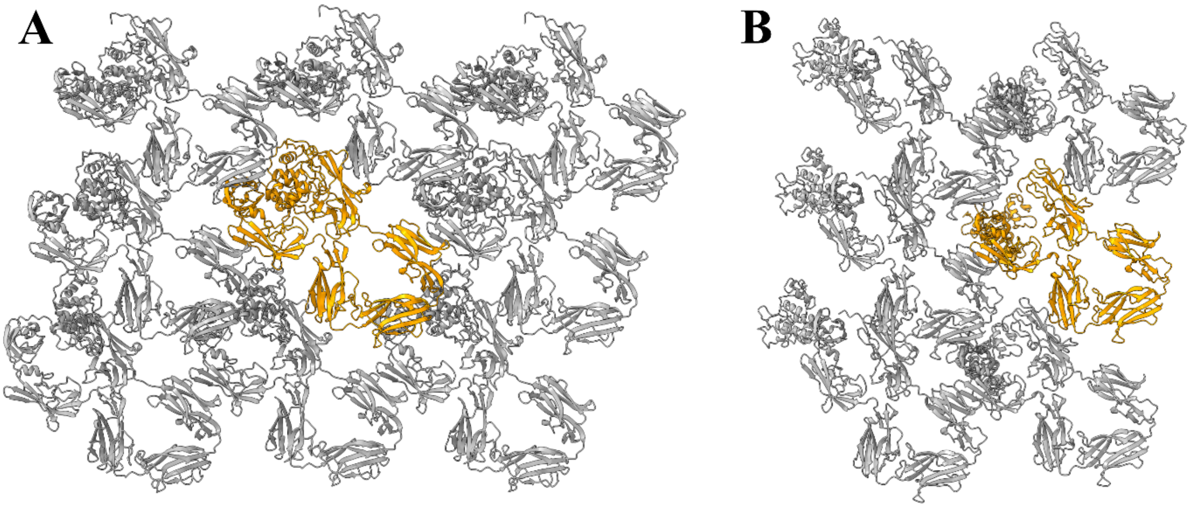

Supplementary Figure 23: Top view of manually built (grey) or partial model provided by SymProFold (orange). A) EA1 from *Bacillus anthracis*. **B**) SLP from *Bacillus licheniformis*.

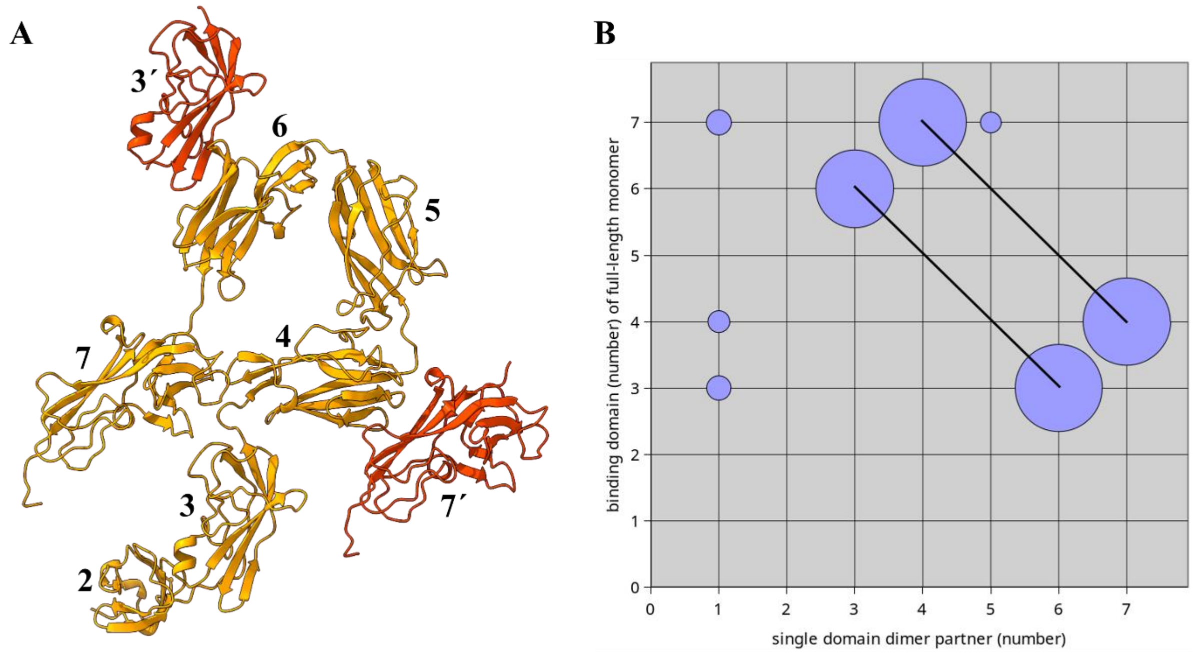

Supplementary Figure 24: Domains of EA1 of *Bacillus anthracis*. A) The strongest interactions were found between domain 4 – 7´ and 6 – 3´. Domain 1 responsible for cell wall binding is not shown. Domains 2-7 are shown in orange, domains 3’ and 7’ in red. **B)** Predicted symmetry complexes of full-length and single domain each. The circle diameters represent the respective ipTM+pTM scores. Identical binding sites are connected by a line. The diagram clearly indicates a binding between domain 4 – 7´ and 6 – 3´.

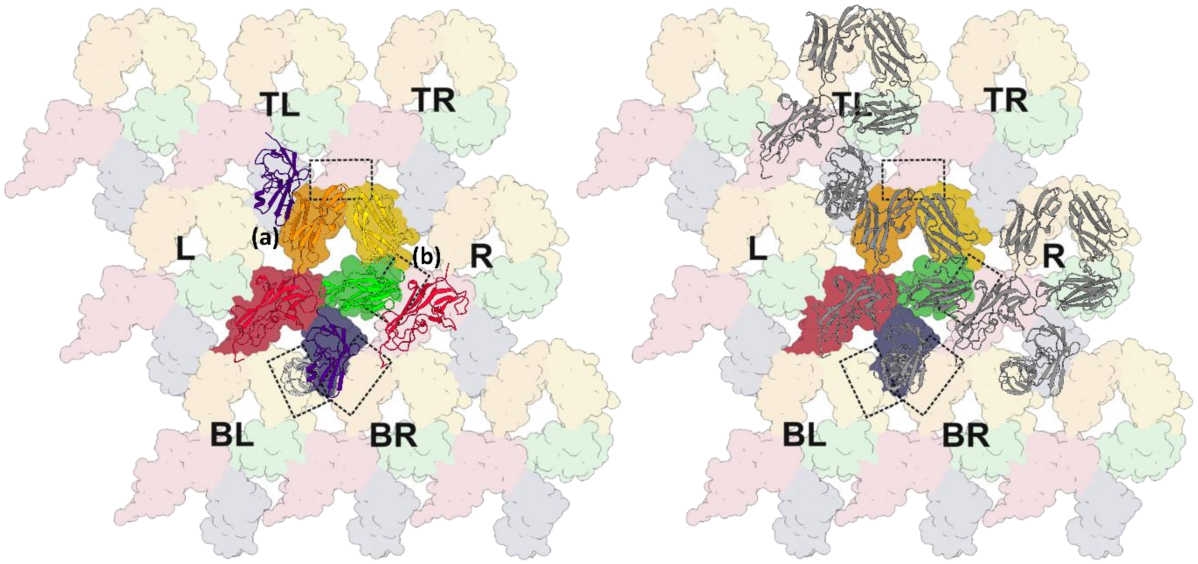

Supplementary Figure 25: Comparison of experimental cryo-EM data of EA1 and calculated SymProFold model. The calculated *p1* SymProFold model (cartoon) of EA1 from *Bacillus anthracis* shows the same two interfaces (a) and (b) as seen in the experimentally derived model (background, surface representation) proposed by Sogues et al. 2023 [2]. The protein domains are colored separately in the cryo EM data (yellow, orange, red, dark blue and green) and marked with letters: L (left), R (right), TR (top right), TL (top left), BR (bottom right), BL (bottom left). The SymProFold model is superimposed in grey. The fully assembled SymProFold model fits the experimental data. The background image was adapted (removed several text descriptions) from Figure 3 of Sogues et al. 2023 [2] and is an open-access article distributed under the terms of the Creative Commons CC BY license [http://creativecommons.org/licenses/by/4.0/].

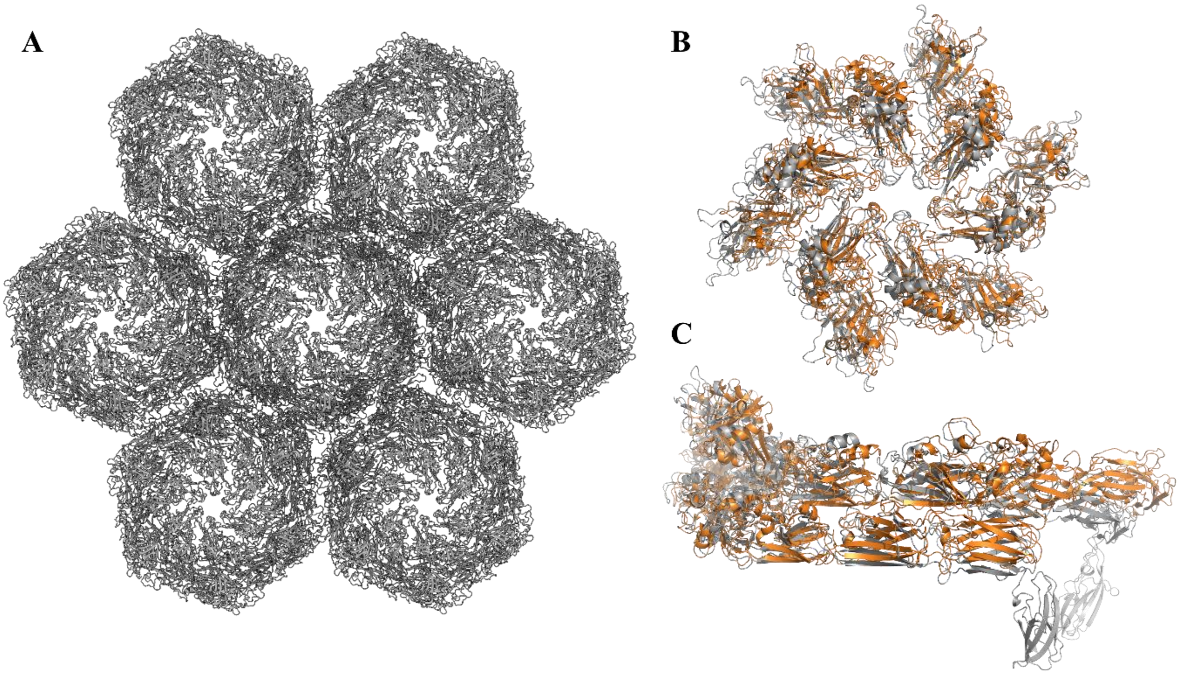

Supplementary Figure 26: Comparison of experimental cryo-EM data of SLP from *Nitrosopumilus maritimus* and calculated SymProFold model. A) Proposed SymProFold model. B) The 6-fold axis of the model (grey) aligns well with the 6-fold axis of the experimental structure of *N. maritimus* (orange, PDB 8C8M [3] [https://doi.org/10.2210/pdb8c8m/pdb]). C) Two chains of the model (grey) aligned with the experimental structure (orange).

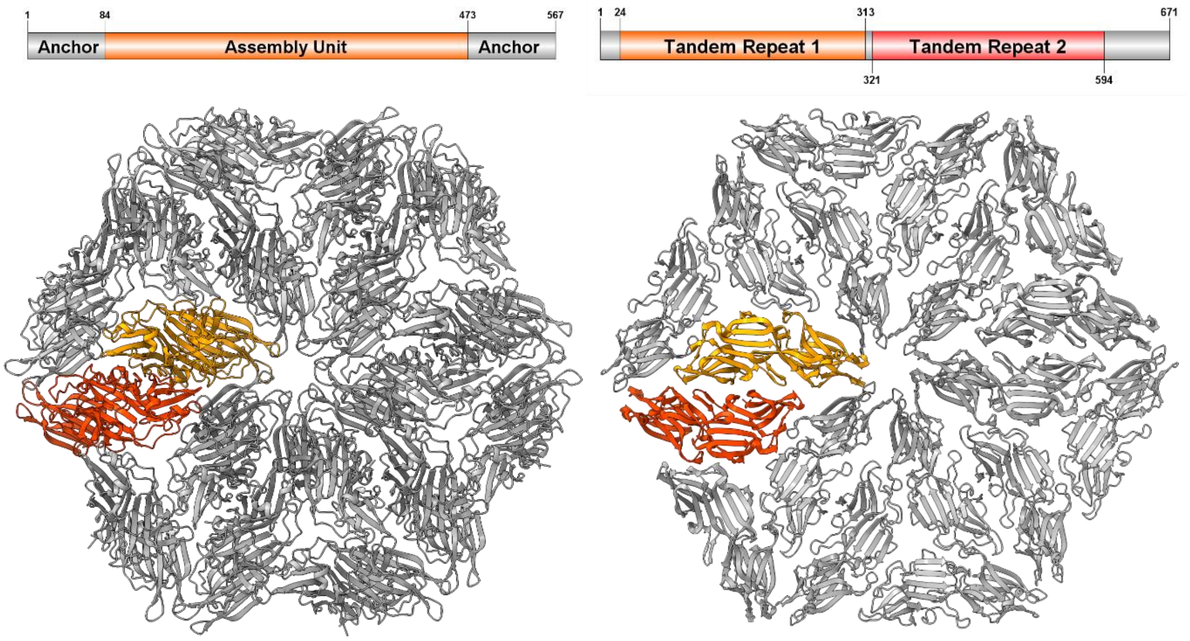

Supplementary Figure 27: Comparison of the calculated SymProFold model of *M. vannielii* (left) and experimental crystal structure of *M. acetivorans* [4] (right). The 2-fold axis of *M. vannielii* is formed by a dimer (domains highlighted in red and orange) compared to the single tandem repeat protein from *M. acetivorans* and *M. vannielii* has an additional N- and C-terminal domain for a putative cell-wall anchor. The overall symmetry, unit cell dimensions, and surface of both species is comparable.

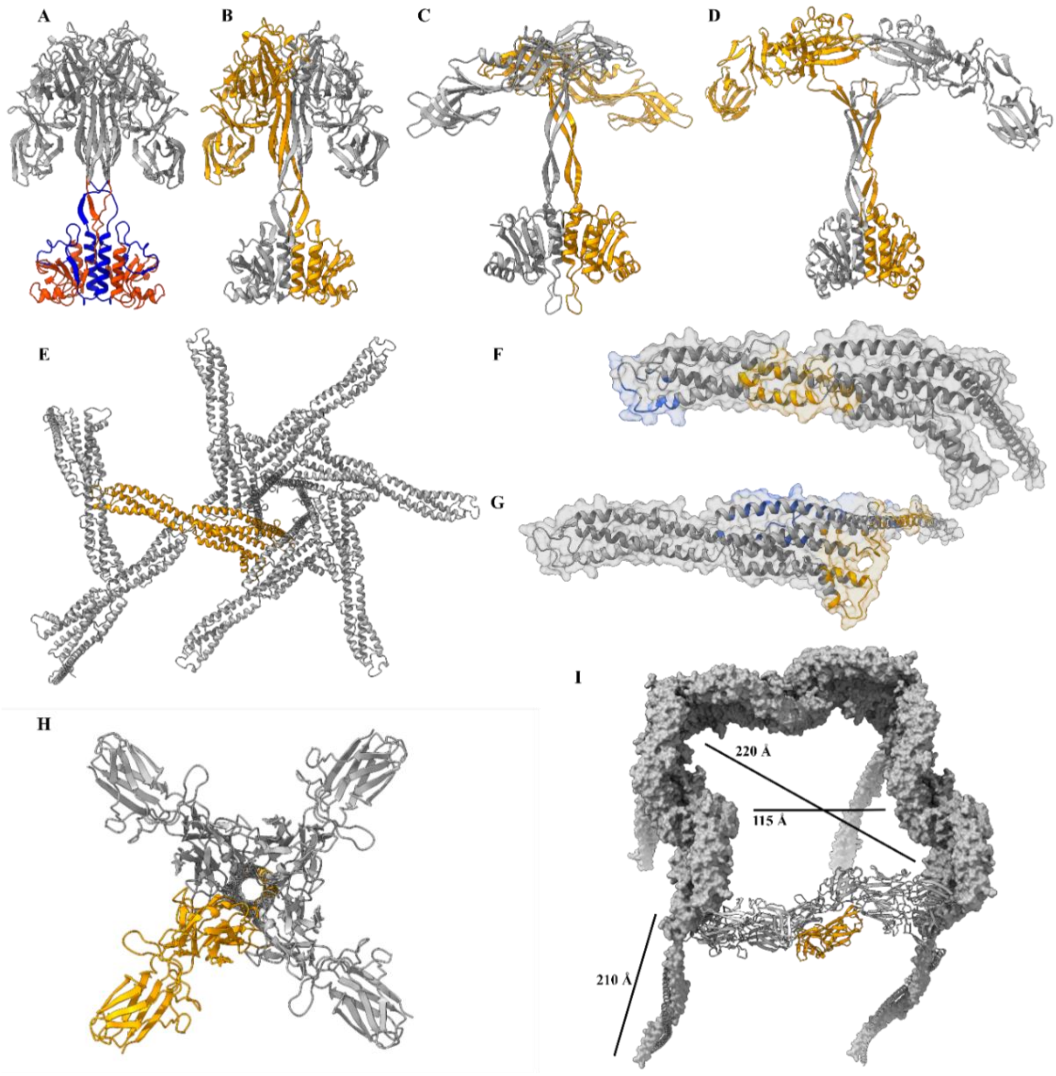

Supplementary Figure 28: Functional analysis of predicted S-layers. A) The *T. thioreducens* S-layer anchor is built up from an intermolecular domain of the N-terminus (blue) and C-terminus (red). **B)** The anchor in the assembly of *M. voltae* comprises two subunits, grey and orange. **C)** Anchor of *T. camini* and **D)** *P. abyssi.* **E)** Top view of the assembly of *C. glutamicum* showing the 3-fold and 6-fold axis. One subunit of *C. glutamicum*, showing the interacting interfaces (blue interacts with orange in the next subunit) of the **F**) 3-fold and **G**) 6-fold axis. **F**) The trimer interface results from a strong interaction between the distal loop (aa216 – aa241, blue) of the 3-fold axis and a distinct groove along the elongated monomer (orange). Several hydrogen bonds, salt bridges, and pi-stacking interactions are observed in this area. **G**) The 6-fold axis is structurally maintained by interactions of the central loop region (orange) with the surface of the adjacent monomer along the C-terminally protruding helix (blue), as well as interactions of the two helices. **H)** Top view of the 4-fold axis of *D. mucosus*. The N-terminal domains 1-5 of the 2-fold arms are not shown. **I)** Fully assembled pore unit of *D. mucosus* and its long anchoring stalk. The dimeric interface is highlighted in orange.

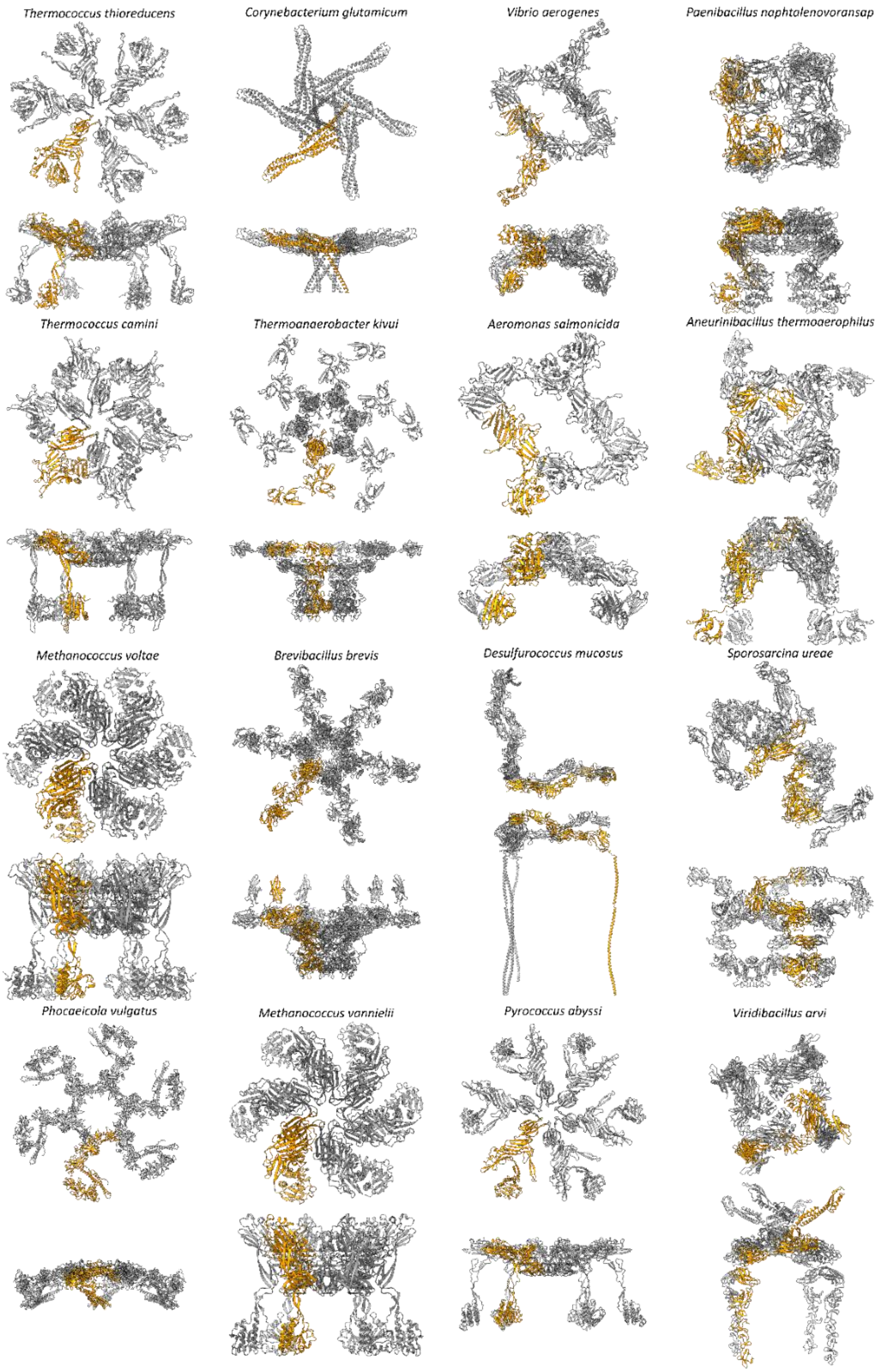

Supplementary Figure 29: Primitive unit cells of the putative models as calculated by SymProFold. The models are shown as primitive unit cells of the calculated S-layer. One subunit is colored orange. Shown in the top view and side view.

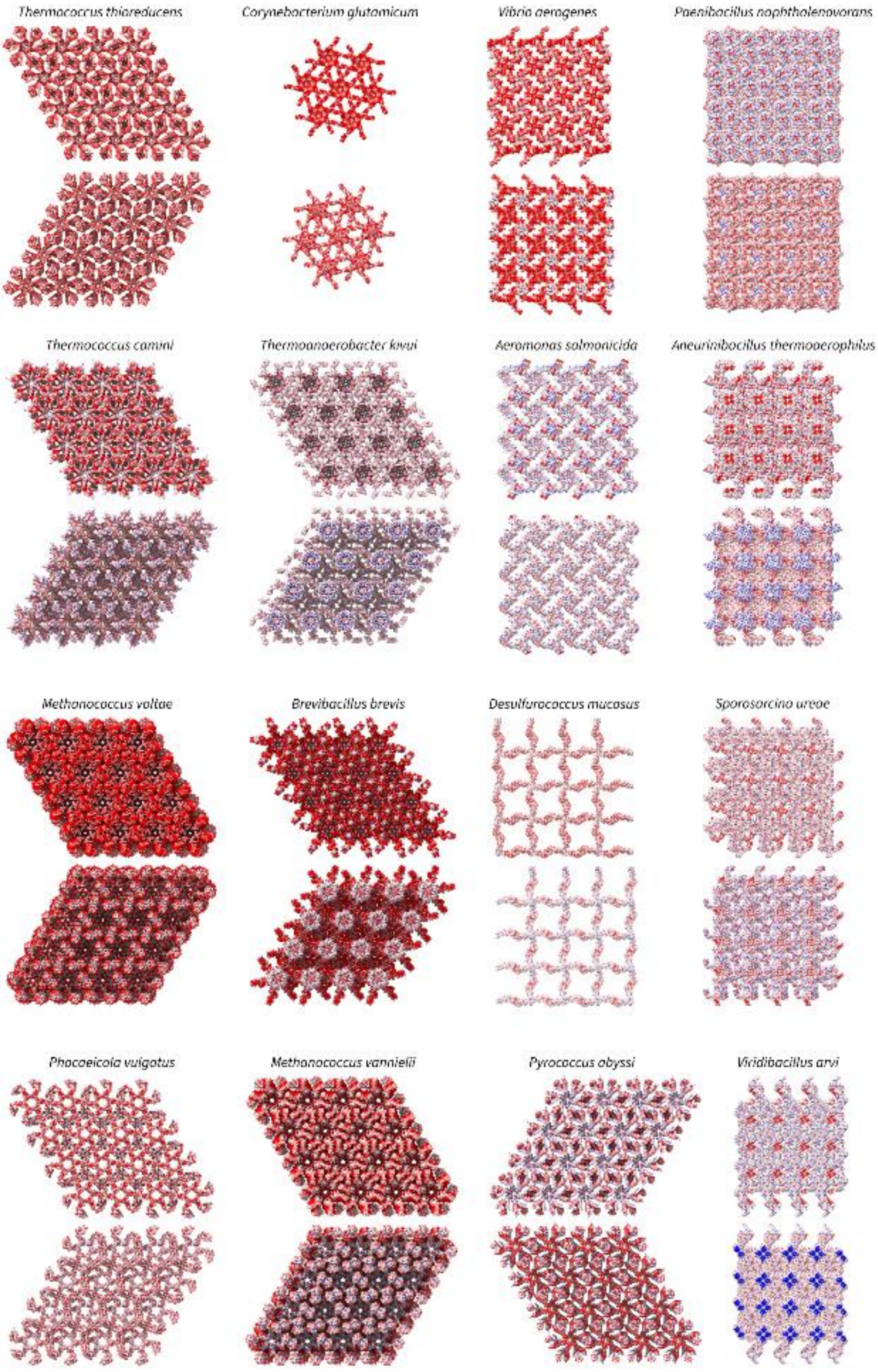

Supplementary Figure 30: Coulombic electrostatic potential of the calculated S-layers primitive unit cell. Coulombic electrostatic potential was calculated with ChimeraX [1]. Colors range from red for negative potential to white for neutral and blue for positive potential. For each S-layer, the side facing the environment (top) and cell wall facing side (bottom) are shown.

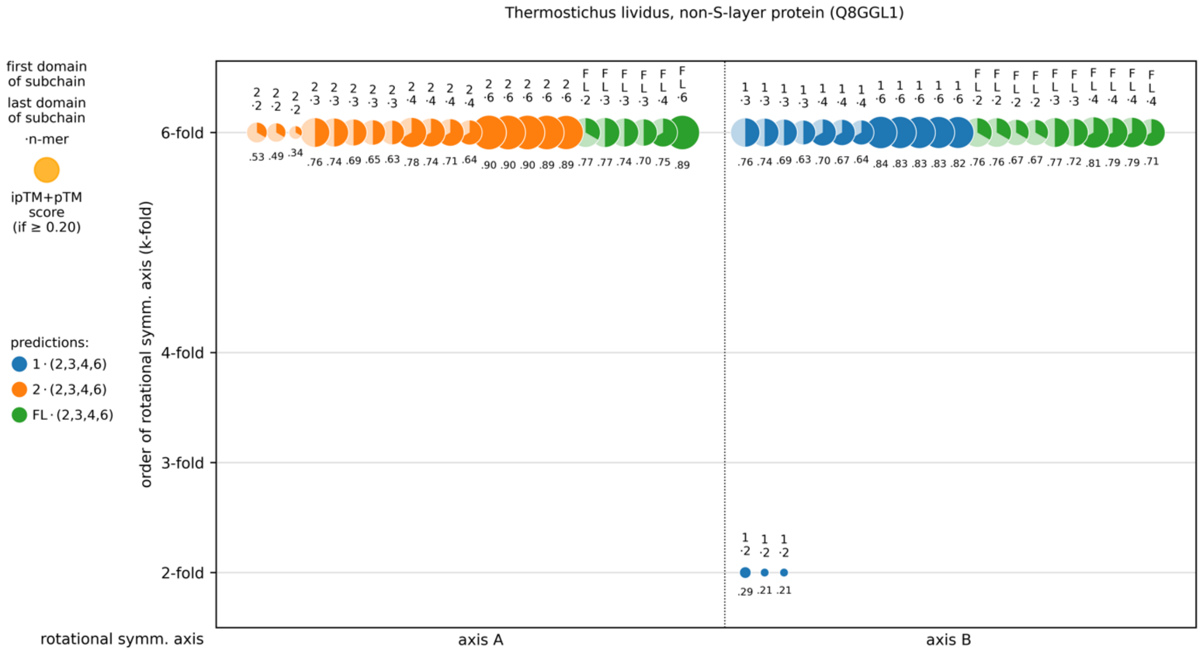

Supplementary Figure 31. Clustering of predicted symmetry complexes for non-S-layer protein KaiC from *Thermostichus lividus* (Q8GGL1). The circle diameters represent the respective ipTM+pTM scores, each color represents a subchain. Axes A and B coincide, spanning of a 2D layer is not possible. Analysis is based on 60 predictions.

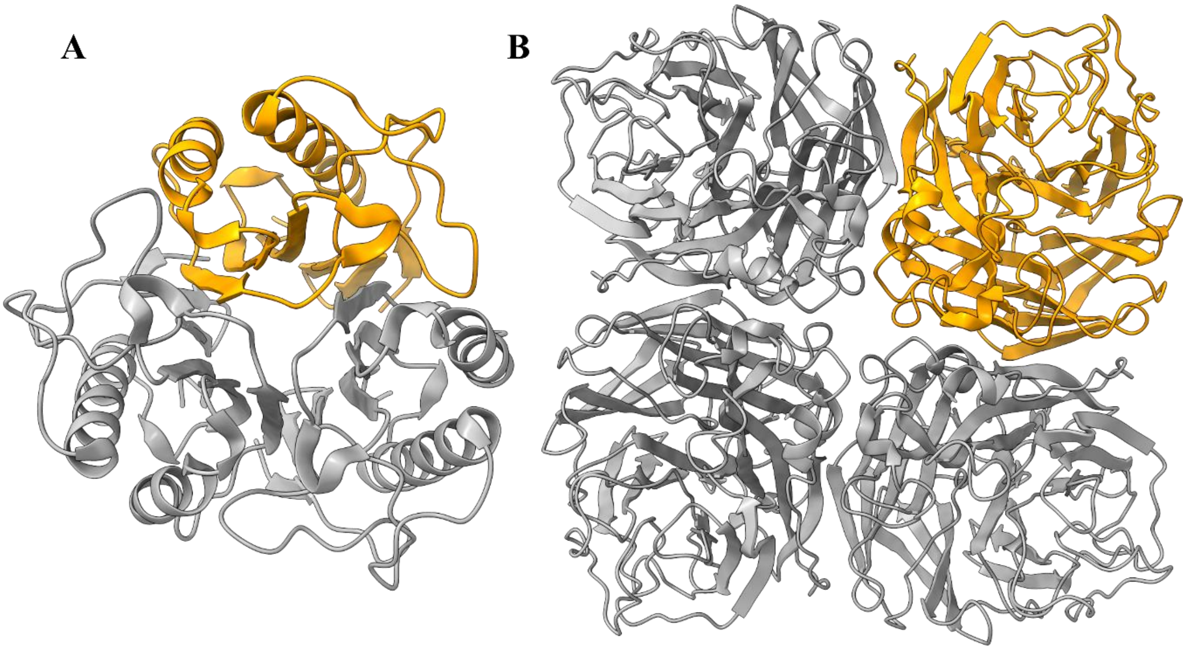

Supplementary Figure 32. Crystal structures of oligomers with only one symmetry axis. A) Crystal structure of trimeric YabJ from Bacillus subtilis (PDB 5Y6U [https://doi.org/10.2210/pdb5y6u/pdb]) [5] and B) N9 neuraminidase from *Influenza A virus* (6MCX) [6]. Models are shown in grey, with one domain highlighted in orange.

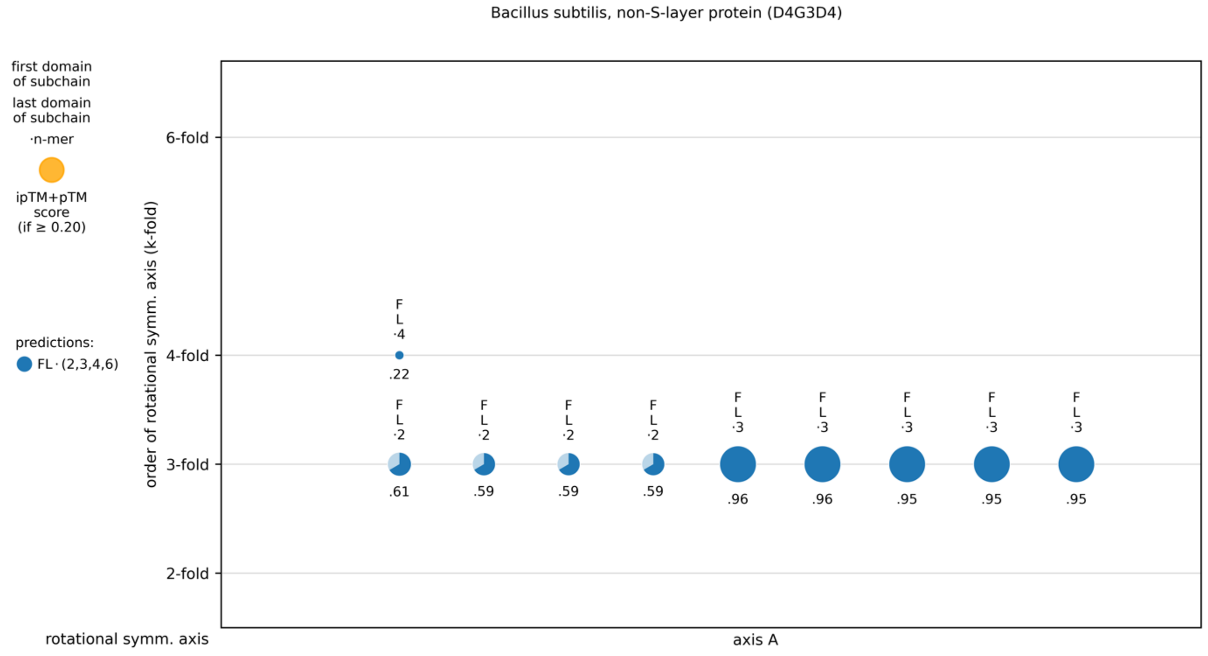

Supplementary Figure 33. Clustering of predicted symmetry complexes for non-S-layer protein YabJ from *Bacillus subtilis* (D4G3D4). The circle diameters represent the respective ipTM+pTM scores. Since there is only one axis of rotational symmetry, spanning of a 2D layer is not possible. Analysis is based on 20 predictions.

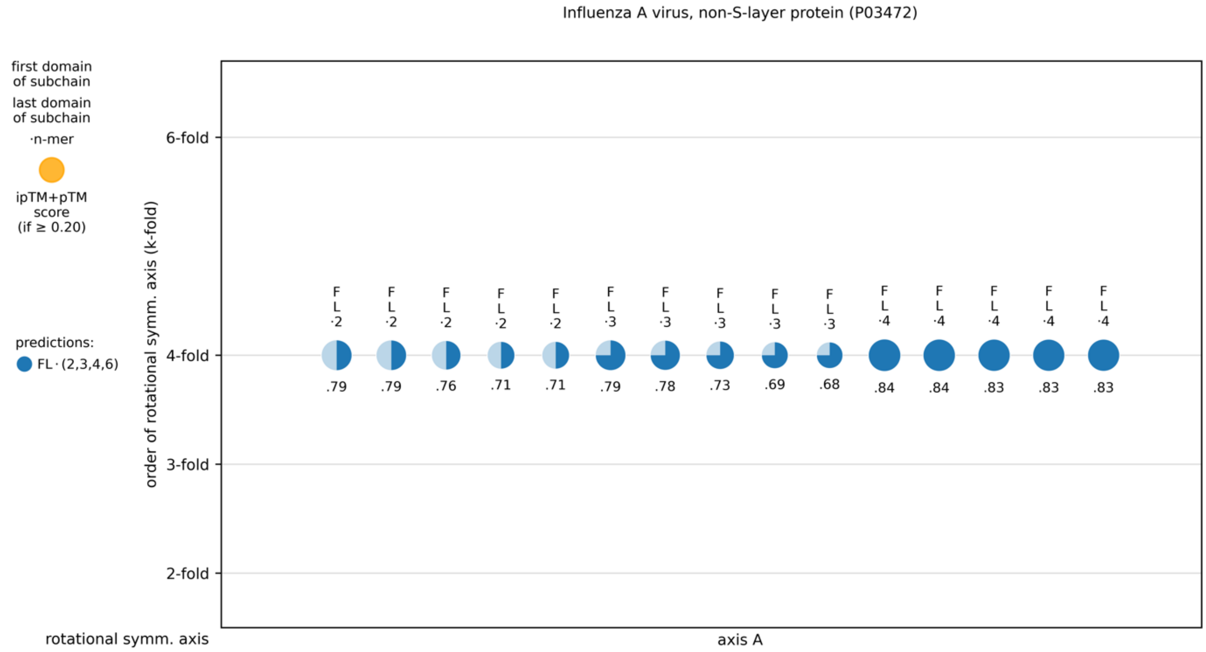

Supplementary Figure 34. Clustering of predicted symmetry complexes for non-S-layer protein N9 neuraminidase from *Influenza A virus* (P03472). The circle diameters represent the respective ipTM+pTM scores. Since there is only one axis of rotational symmetry, spanning of a 2D layer is not possible. Analysis is based on 20 predictions.

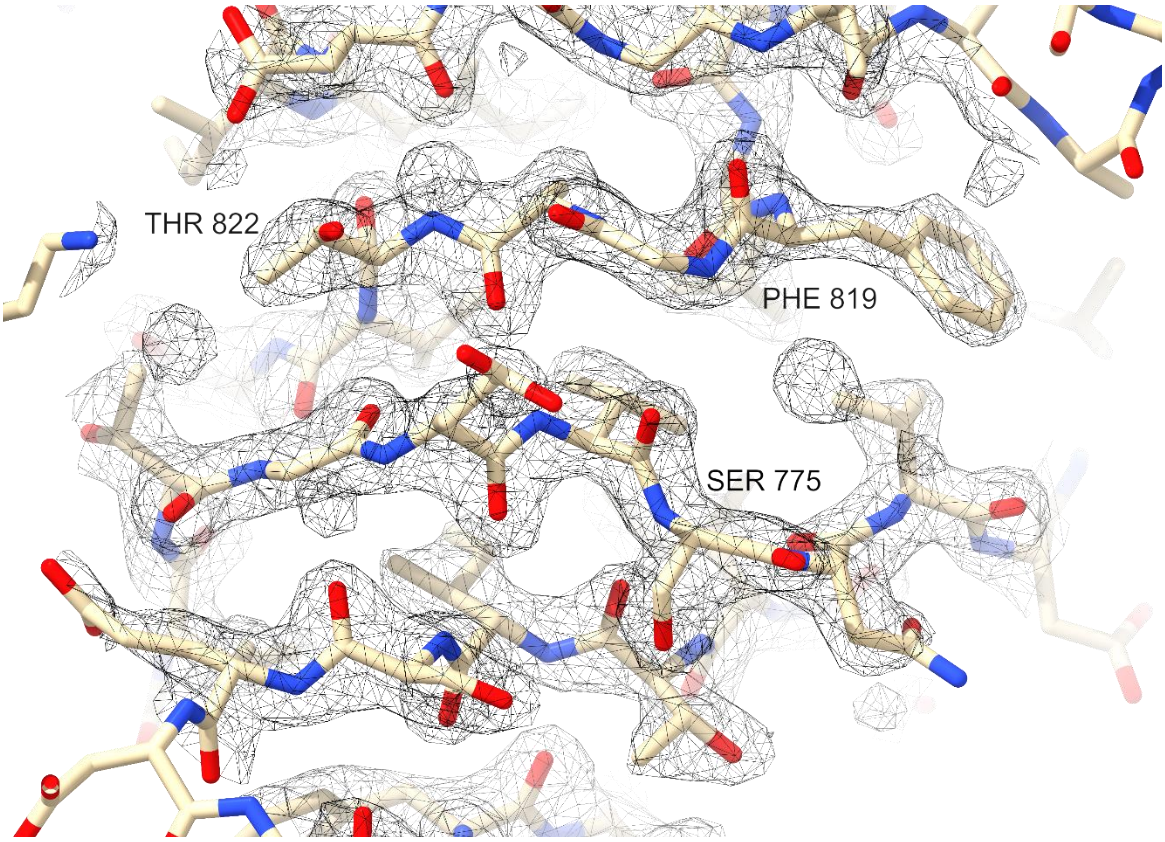

Supplementary Figure 35. Exemplary structure region of 9FS9 ($Varv_{765-844}$) with electron density (2Fo-Fc map at RMS contour level 1.5), residues S775, F819, and T822 of chain A are labeled for orientation. The image was prepared with ChimeraX [1].

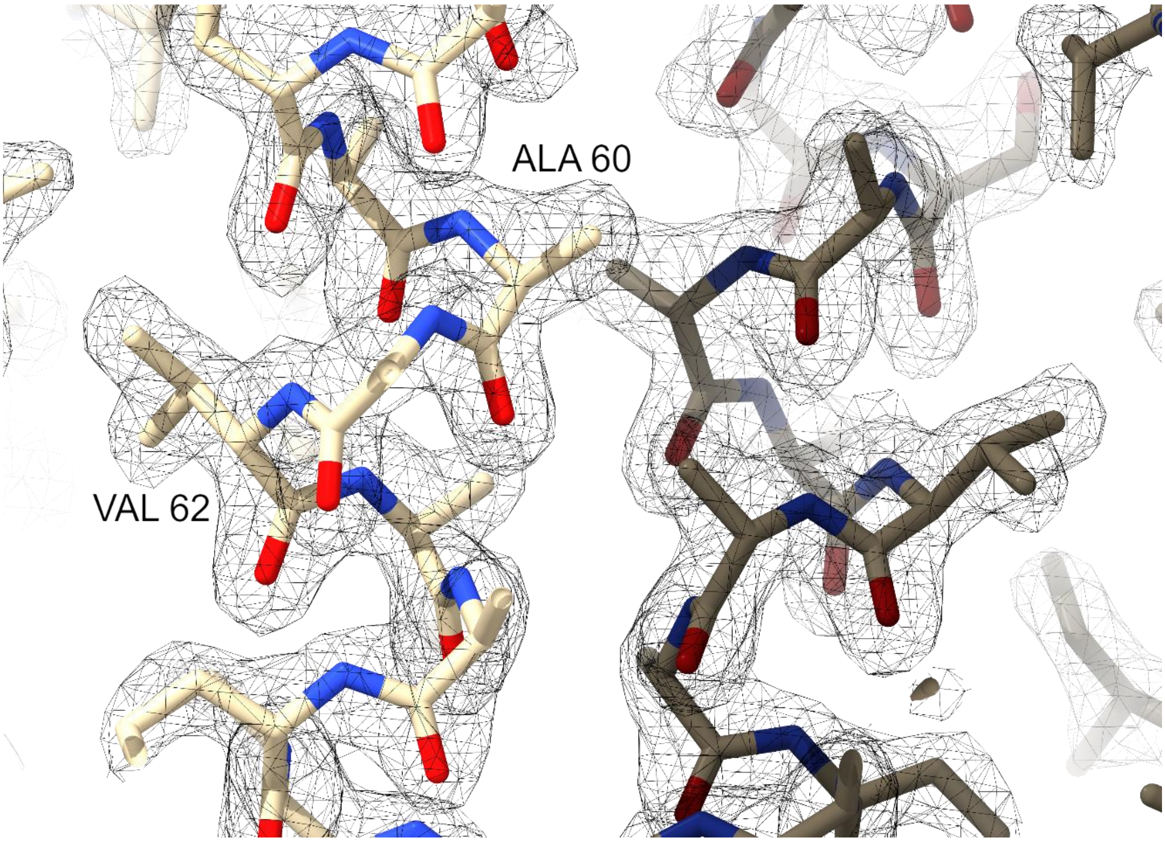

Supplementary Figure 36. Exemplary structure region of 9FSA ($Mvol_{24-75/484-576}$) with electron density (2Fo-Fc map at RMS contour level 1.5) Residues A60 and V62 were labeled for orientation, the symmetry mate is depicted in a darker color. The image was prepared with ChimeraX [1].

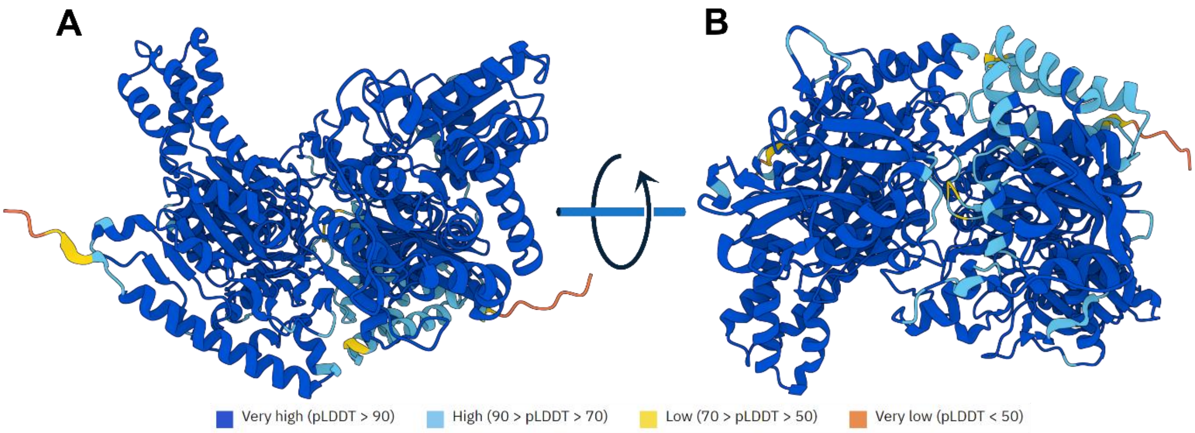

Supplementary Figure 37. AlphaFold model of *Photobacterium gaetbulicola* [https://www.uniprot.org/uniprotkb/A0A0C5WNU2/entry] **colored according to the plDDT score.** Obtained from [https://alphafold.ebi.ac.uk/entry/A0A0C5WNU2] and last updated 2022-11-01 with AlphaFold Monomer v2.0. B is rotated 180 ° compared to A as indicated by the arrow.

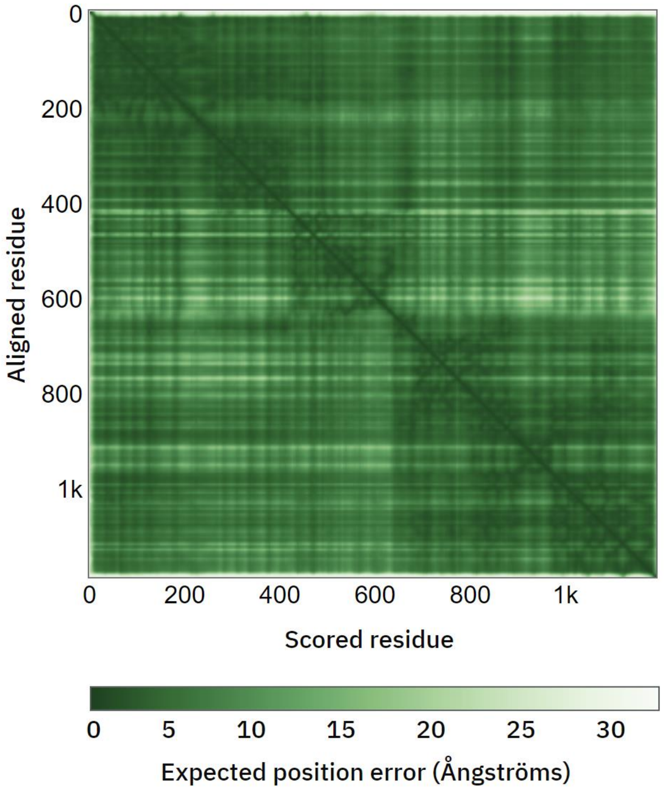

Supplementary Figure 38. Predicted aligned error (PAE). PAE of the predicted AlphaFold structure of A0A0C5WNU2 [https://alphafold.ebi.ac.uk/entry/A0A0C5WNU2].

Supplementary Table 1. Available experimental structures of assembled S-layers and fragments of SLPs. Species names are italicized.

Organism	Protein / Accession	PDB Code	Note	Reference
**Gram-positive**				
Lactobacillus acidophilus	SlpA (P35829)	7QLD, 7QFL, 7QLE, 7QFG, 8ALU, 8BT9	Crystal structures of fragments	[7]
Lactobacillus acidophilus	SlpX (Q5FLN0)	7QFI, 7QFJ, 7QFK, 8AOL	Crystal structures of fragments	[7]
Lactobacillus amylovorus	SlpA (E4SK47)	7QLH, 7QEC, 7QEH, 8Q1O	Crystal structures of fragments	[7]
Geobacillus stearothermophilus	SbsB (Q45664)	4AQ1	Assembly proposed based on EM projection map	[8]
Geobacillus stearothermophilus	SbsC (O68840)	2RAI, 4UIC, 4UID, 4UIE, 4UJ6, 4UJ7, 4UJ8, 5FTX, 5FTY	Crystal structures of fragments	[9–12]
Bacillus anthracis	Sap (P49051)	3PYW, 6BT4, 6QX4, 6HHU	Crystal structures of fragments	[13–15]
Bacillus anthracis	EA1	8OPR		[2]
Clostridium difficile	SlpA (Q183M8)	3CVZ, 7ACV, 7ACW, 7ACX, 7ACY, 7ACZ, 7QGQ, 8BBY	Full model, assembly seen in X-ray structures, fits to cryoET data	[16,17]
**Gram-negative**				
Deinococcus radiodurans	SlpA (Q9RRB6)	7ZGX, 7ZGY, 8AE1, 8ACQ, 8AGD		[18,19]
Deinococcus radiodurans	HPI (P56867)	8CKA		[20]
Caulobacter cresentus (Caulobacter vibrioides)	RsaA (P35828)	5N8P, 6P5T, 6T72, 6Z7P, 7PEO, 8BQE, 5N97	Full model, assembly seen in X-ray structures, fits to cryoET data	[21–24]
**Archaea**				
Haloferax volcanii (Halobacterium volcanii)	csg (P25062)	7PTP, 7PTR, 7PTT, 7PTU	Full model, cryoEM, cryoET	[25]
Methanosarcina mazei	ORF492 (Q50245)	1L0Q		[26]
Methanosarcina acetivorans	Major S-layer protein (Q8TSG7)	3U2H, 3U2G	Full model, Assembly seen in X-ray structures	[4]
Sulfolobus acidocaldarius	SlaA (Q4J6E5)	8AN2, 8AN3, 7ZCX	Full model, cryoEM, cryoET	[27]
Sulfolobus acidocaldarius	SlaA/SlaB	8QOX		[28]
Nitrosopumilus maritimus	A9A4Y9	8C8R, 8C8L, 8C8M, 8C8N, 8C8K, 8C8O	cryoEM	[3]

Supplementary Table 2. Homology analysis of the presented 18 test cases by RCSB sequence similarity (using MMseqs2 [29]) search and HHpred [30]. RCSB sequence similarity search outputs matching results for only four out of the 18 test cases. HHpred analysis shows that only 9-26% of the input sequences could be aligned. Species names are italicized.

		**RCSB sequence similarity search**			**HHpred**			
Organism	Uni Prot ID	Best Hit UniProt ID	SS %	Aligned region	Best Hit	E-value	Aligned region	Input sequence
C. glutamicum	Q2VRQ3	No result			7WOO_I	80	11%	1-491 (FL)
A. salmonicida	P35823	No result			6P1E_B	3	16%	22-502 (*)
V. quintilis	A0A1M7YYM3	No result			6P1E_B	2.1	20%	22-642 (*)
V. aerogenes	A0A1M5ZCF8	No result			6P1E_B	1.8	19%	22-646 (*)
T. camini	A0A7G2D8K1	No result			3U2G_A	0.053	12%	1-489 (FL)
P. vulgatus	A0A5P3AY64	No result			4ZXQ_D	0.011	13%	1-1100 (FL)
M. vannielii	A6URZ5	No result			3U2G_A	0.0054	19%	1-567 (FL)
T. thioreducens	A0A0Q2M111	No result			3U2G_A	0.00035	15%	1-607 (FL)
V. arvi	A0A0K2Z0V7	No result			4UIC_A	0.000034	25%	1-1016 (FL)
T. kivui	P22258	3PYW	27%	5-129	8ACQ_A	0.000012	26%	1-762 (FL)
B. brevis	P06546	6CWN	29%	1-172	6CWM_A	1.2e-7	23%	1-1053 (FL)
D. mucosus	E8R795	No result			7PTR_E	9.9e-8	11%	1-904 (FL)
A. thermoaerophilus	Q6TL21	No result			5H3K_A	5.7e-8	9%	1-738 (FL)
D. amylolyticus	I3XTG6	No result			7PTR_E	4.7e-11	15%	1-898 (FL)
M. voltae	Q50833	No result			3U2G_A	4.5e-13	19%	1-576 (FL)
S. ureae	Q0VJW4	8BYS	37%	9-78	4UIC_A	1.1e-15	15%	1-1097 (FL)
P. abyssi	Q9V0N3	No result			3U2G_A	2.7e-17	18%	1-604 (FL)
P. naphthalenovorans	A0A0U2M877	6CWC	34%	1-172	4UIC_A	8.5e-37	15%	1-1053 (FL)

(*) without signal sequence

Supplementary Table 3: Predefined sets of subchains covering different parts of the full-length protein. *x* is the number of amino acids that cover a full domain as defined in the fasta input.

Subchain name	Subchain definition	Example with 6 domains	Example with 3 domains	Predefined subchain set
full-length	Full-length (FL) sequence	[i]-[ii]-[iii]-[iv]-[v]-[vi]	[i]-[ii]-[iii]	standard
w/o N-terminus	Removal of 10 % + *x* (sequence length) from the N-terminus.	[ii]-[iii]-[iv]-[v]-[vi]	[ii]-[iii]	standard
w/o C-terminus	Removal of 10 % + *x* (sequence length) from the C-terminus.	[i]-[ii]-[iii]-[iv]-[v]	[i]-[ii]	standard
first 1/3	First third of domains.<br>At least 20 % + *x* (sequence length) from the N-terminus.	[i]-[ii]	[i]	standard
last 1/3	Last third of domains.<br>At least 20 % + *x* (sequence length) from the C-terminus.	[v]-[vi]	[iii]	standard
middle 1/3	Middle third of domains.<br>Full length less “first 1/3” and “last 1/3”.	[iii]-[iv]	[ii]	extended

Supplementary Table 4: Symmetry axes identified by full-length predictions. Full-length predictions can lead to an assembly model, but generally, subchain predictions are necessary. Reasons include A) one strongly favored symmetry center, B) too large system sizes, and C) twisted assemblies to produce both symmetry centers in one prediction. Species names are italicized. For green-marked proteins both symmetry axes could be identified successfully.

Organism Accession	Symmetry axes automatically identified by full-length predictions			Reason why not both symmetry axes can be automatically identified by full-length predictions			
	0 of 2	1 of 2	2 of 2	A) large system size	B) one strongly favored	C) twisted assembly	other
C. glutamicum Q2VRQ3			X				
A. salmonicida P35823		X			X		
V. quintilis A0A1M7YYM3	X (*)						X
V. aerogenes A0A1M5ZCF8		X			X		
T. camini A0A7G2D8K1		X	X (**)			X	
P. vulgatus A0A5P3AY64		X		X (6600aa)			
M. vannielii A6URZ5		X	X (**)			X	
T. thioreducens A0A0Q2M111		X					X
V. arvi A0A0K2Z0V7	X					X	
T. kivui P22258		X		X (4572aa)			
B. brevis P06546		X		X (6318aa)			
D. mucosus E8R795		X		X (3616aa)			
A. thermoaerophilus Q6TL21			X				
D. amylolyticus I3XTG6		X		X (3592aa)			
M. voltae Q50833		X	X (**)			X	
S. ureae Q0VJW4		X		X (4388aa)			
P. abyssi Q9V0N3		X					X
P. naphthalenovorans A0A0U2M877	X			X (4212aa)			

(*) half of the 4-fold axis is predictable by FL·2

(**) 2 of 2 can be seen in the assembly model, but no automated identification

Supplementary Table 5: ipTM+pTM scores of the predicted S-layer models published in this study. Data basis are complete symmetry complexes (models) that show the predicted order of symmetry. For each axis cluster, the highest-ranking model is included in the statistics.

Organism	UniProt ID	Axis A cluster ipTM+pTM	Axis B cluster ipTM+pTM
D. mucosus	E8R795	0.77	0.75
D. amylolyticus	I3XTG6	0.78	0.74
S. ureae	Q0VJW4	0.57	0.67
A. salmonicida	P35823	0.89	0.88
V. aerogenes	A0A1M5ZCF8	0.88	0.87
V. quintilis	A0A1M7YYM3	0.89	0.88
A. thermoaerophilus	Q6TL21	0.88	0.84
V. arvi	0A0K2Z0V7	0.80	0.41
P. naphthalenovorans	A0A0U2M877	0.91	0.21 (*)
M. vannielii	A6URZ5	0.85	0.76
M. voltae	Q50833	0.82	0.75
T. camini	A0A7G2D8K1	0.75	0.61
T. thioreducens	A0A0Q2M111	0.79	0.45
P. abyssi	Q9V0N3	0.77	0.69
T. kivui	P22258	0.80	0.74
B. brevis	P06546	0.81	0.74
P. vulgatus	A0A5P3AY64	0.55	0.57
C. glutamicum	Q2VRQ3	0.65	0.58
Median		0.80	0.74

(*) Axis C cluster, because clusters A and B coincide

Supplementary Table 6: Data collection and refinement statistics of PDB 9FSD9 [https://doi.org/10.2210/pdb9fs9/pdb] **and PDB 9FSA** [https://doi.org/10.2210/pdb9fsa/pdb]**.** Statistics for the highest resolution shell are given in parentheses

	9FS9: $Varv_{765-844}$	9FSA: $Mvol_{24-75/484-576}$
**Data collection**		
Wavelength (Å)	1.3414	1.3414
Space group	$P\ 2_1$	$P\ 2\ 2_1\ 2_1$
Cell dimensions		
a, *b*, *c* (Å)	68.0, 35.0, 68.0	46.3, 53.3, 55.6
α, β, γ (°)	90, 91.2, 90	90, 90, 90
Resolution (Å)	48.6 - 2.1 (2.18 - 2.1)	46.3 - 2.05 (2.12 - 2.05)
R_{merge}	0.116 (0.808)	0.322 (1.80)
$I / \sigma I$	9.3 (1.4)	8.2 (1.6)
Completeness (%)	99.8 (100.0)	90.7 (70.1)*
Redundancy	6.0 (4.6)	23.5 (15.8)
CC ½	0.994 (0.589)	0.996 (0.560)
**Refinement**		
No. reflections	19156	4977
R_{work}	0.197	26.5
R_{free}	0.248	28.2
No. atoms	2699	942
Protein	2535	928
Ligand/ion	1	0
Water	163	14
B-factors	41.01	23.51
Protein	41.19	23.67
Ligand/ion	35.81	-
Water	38.30	12.90
R.m.s. deviations		
Bond lengths (Å)	0.009	0.008
Bond angles (°)	1.59	1.19
Ramachandran		
Favored (%)	97.51	95.42
Allowed (%)	2.49	4.58
Outliers (%)	0.00	0.00
Rotamer outliers (%)	3.10	2.15
Clashscore	4.20	5.83

* Ellipsoidal completeness is given from Staraniso [31]

Supplementary Table 7: Number of effective sequences (Neff) [32] for MSAs of different subchains. The integrated Neff values were calculated from merged individual MSAs (bfd, mgnify, uniref90) after removing duplicates. Neff values were determined with NEFFy [33]. Column 'subchain': Data basis are subchains of complete symmetry complexes (models) that show the predicted order of symmetry. For each axis cluster (A and B) of a species, the subchain of the highest-ranking model is shown. Column 'ipTM+pTM': For the respective subchain, the ipTM+pTM value of the highest-ranking model with the predicted order of symmetry is shown.

**Species UniProt ID**	**Sub-chain**	**bfd Neff**	**bfd depth**	**mgnify Neff**	**mgnify depth**	**uniref90 Neff**	**uniref90 depth**	**integrated Neff**	**integrated depth**	**ipTM+pTM**
D. mucosus	17	108.1	4151	1.2	737	0.9	59	108.9	4379	0.77
E8R795	68	12.1	747	13.9	500	46.1	1353	68.6	2548	0.75
D. amylolyticus	17	147.2	4648	1.2	115	0.9	42	147.4	4802	0.78
I3XTG6	68	11.2	644	17.5	500	41.8	1111	66.7	2211	0.74
S. ureae	68	6.8	196	0.8	26	2.2	56	8.9	275	0.57
Q0VJW4	28	6.3	390	1.4	115	5.2	196	10.6	699	0.67
A. salmonicida	12	1.8	397	0.2	5	2.2	89	3.6	488	0.89
P35823	3	1.3	21	0.2	2	1.6	49	1.8	63	0.88
V. aerogenes	3	1.1	21	0.2	2	1.6	49	1.7	64	0.88
A0A1M5ZCF8	12	1.7	71	0.3	7	1.4	122	2.7	199	0.87
V. quintilis	3	1.1	21	0.3	3	1.6	49	1.8	65	0.89
A0A1M7YYM3	12	1.5	68	0.3	8	1.4	134	2.6	206	0.88
A. thermoaerophilus	34	63.4	3028	18.4	500	18.7	3805	86.0	7273	0.88
Q6TL21	13	56.5	2202	11.9	500	10.8	335	77.4	3028	0.84
V. arvi	28	8.5	2116	1.4	118	5.8	218	14.8	2444	0.80
A0A0K2Z0V7	13	25.0	2997	2.6	500	6.3	504	31.0	3842	0.41
P. naphthalenovorans	68	26.1	628	1.6	31	6.2	144	32.7	803	0.91
A0A0U2M877	13	150.1	3670	10.3	500	120.7	9999	120.7	9913	0.21
M. vannielii	12	62.6	2306	1.6	95	2.2	115	64.8	2508	0.85
A6URZ5	FL	21.4	3038	1.4	208	2.5	253	23.7	3467	0.76
M. voltae	12	53.7	2408	1.3	88	2.5	112	56.0	2601	0.82
Q50833	FL	20.5	3169	1.4	181	3.0	251	23.2	3586	0.75
T. camini	FL	3.4	1486	0.4	8	1.2	37	3.9	1527	0.75
A0A7G2D8K1	23	4.2	1366	0.4	15	1.2	35	4.7	1414	0.61
T. thioreducens	FL	20.5	3379	0.4	268	2.4	345	22.3	3942	0.79
A0A0Q2M111	12	58.3	2211	0.4	97	2.4	160	60.2	2452	0.45
P. abyssi	FL	16.5	3899	0.6	184	3.4	312	19.6	4368	0.77
Q9V0N3	12	59.9	2150	0.4	137	2.8	202	62.2	2469	0.69
T. kivui	29	71.7	1925	0.2	4	1.0	25	72.4	1952	0.80
P22258	13	169.7	3411	7.4	500	84.9	9999	255.4	13670	0.74
B. brevis	13	132.8	3502	10.7	500	33.8	9999	165.4	13845	0.81
P06546	29	50.1	2086	0.4	11	1.2	44	50.5	2138	0.74
P. vulgatus	45	9.4	320	4.1	500	2.1	69	13.0	764	0.55
A0A5P3AY64	25	9.0	611	1.4	393	1.1	136	10.5	1126	0.57
C. glutamicum	FL	1.3	49	1.1	65	1.1	64	2.4	166	0.65
Q2VRQ3										

Supplementary Method 1: **Domain Identification using Domain_Separator**

We tested Domain_Separator on 500 randomly selected structures between 800 and 2000 amino acids from the AlphaFold Protein Structure Database [34]. In Figure S1, the protein A0A0C5WNU2 is shown as an example. The Domain_Separator tool identifies upon several iterations different domains of a protein using the protein coordinate file as input. Each identified domain is colored separately as shown in Figure S1B. The linker regions between the domains are colored in red (Figure S1B) and are cut after the amino acid highlighted in dark blue (circled in Figure S1B). The resulting sequences of the individual domains are written in a fasta file output in separated lines (Figure S1C). This file can be used as the direct input for SymProFold. The tested sequences correlate to manually split domains and Domain_Separator achieved comparable results, with slight differences in linker length. The fasta file output from Domain_Separator can still be manually modified before starting SymProFold.

Supplementary Method 2: **Subchain Sets**

A set of subchains is defined, each subchain covering a different range of domains of the protein (Table S3). All subchains start and end at previously defined domain boundaries. The *standard subchain set* includes 5 subchains, including the full-length sequence, a subchain without the N-terminus, a subchain without the C-terminus, the first third of the domains, and the last third of the domains. To mitigate the effects of very large or small domains, minimum lengths were defined. The *extended subchain set* additionally contains a subchain with the middle third of domains.

Supplementary Method 3: **Prediction of Oligomer Sets and Filtering**

The subchain predictions are filtered by clashes determined by the function command *clashes* in ChimeraX [1] with standard values for *overlap cutoff* and *hbond allowance*. No further weighting by prediction confidence (pLDDT) was performed. The overlap between 2 atoms *i* and *j* with distance d_{ij} is calculated as shown in Supplementary Equation (1).

$$\mathrm{overlap_{ij}} = \mathrm{r_{VDWi}} + \mathrm{r_{VDWj}} - \mathrm{dd_{ij}} - \mathrm{allowance_{ij}} \quad (1)$$

allowance: "allowance for potentially hydrogen-bonded pairs" [1]

Protein chains passing incorrectly through the neighboring molecule and forming falsely intermolecular β-sheets are a problem that occasionally occurs in AlphaFold-Multimer predictions (0.1%-1% of the calculations carried out as part of this study). SymProFold contains a built-in algorithm that automatically excludes such predictions by detection of unusually high fractions of intermolecular β-sheets. This filter can also be deactivated if intermolecular β-sheets are expected.

Algorithm for defining the C-terminus:

First, for each residue in a chain, the error estimate (RMSD) is calculated from its respective lDDT using the formula in [35]. Then, the C-terminus includes all residues starting from the C-terminal end (in the upstream direction) that have a RMSD > 5Å, up to the first with an RMSD <= 5Å, which is no longer assigned to the C-terminus.

Algorithm for defining the N-terminus:

Part 1: The N-terminus includes all residues starting from the N-terminal end (in the downstream direction) that have a RMSD > 5Å, up to the first with an RMSD <= 5Å, which is no longer assigned to the N-terminus.

A signal sequence with an α-helical part having RMSD <= 5Å would result in the loose section between the α-helical part and the first domain not being included in the N-terminus. To also include this section, the following part 2 of the algorithm is applied:

Part 2: If the length of the N-terminus according to part 1 is <= 20 and if all 5 residues between residue 21 and 25 have a RMSD > 5Å, all residues in the range 1-21 are included into the N-terminus. Additionally, all residues starting from residue 21 that have an RMSD > 5Å are included, up to the first with an RMSD <= 5Å, which is no longer assigned to the N-terminus.

Supplementary Method 4: Clustering via Interfaces

Calculation of Interface Matrix

For all individual symmetry complexes, an interface matrix D is created. The matrix elements d_{ij} represent the distances between the interface residues i, j of two adjacent monomers of the symmetry complex.

The interface residues are determined using the command *interface* in the ChimeraX [1] function library. The indices of each matrix element d_{ij} correspond to the residue indices *i*, *j* of both monomers. Values of the matrix elements d_{ij} are the intermolecular C_α atom distances in Ångstrom (Å). Matrix elements between non-interface residues or distances larger than a cutoff value of 10 Å are set to zero, as well as matrix elements denoted by at least one empty residue or at least one residue of a N- or C-terminus.

Calculation of Correlation Coefficient

Interface matrices as defined in this study are generally asymmetric but can be symmetric in special cases. The order of the axes in both interface matrices D^k and D^l can be different reflecting the exchangeable order of the underlying interface monomers. Therefore, correlations between two interface matrices D^k and D^l must be determined for both possible pairwise transposition states (D^k, D^l) and $\left(D^k, D^{l^{\mathrm{T}}}\right)$. For the same transposition state of both matrices D^k and D^l, the correlation $\mathrm{corr}(D^k, D^l)$ is calculated as the sum of all ratios of twice non-zero matrix elements d^k_{ij} and d^l_{ij} with a scaling factor (Supplementary equation (2)). The indices *k*, *l* label different symmetry complexes (nodes), the indices *i*, *j* are the residue indices within the respective monomers.

$$\mathrm{corr}(D^k, D^l) = \frac{1}{n}\sum_{d^k_{ij} \neq 0 \,\wedge\, d^l_{ij} \neq 0} \frac{\min\left(d^k_{ij}, d^l_{ij}\right)}{\max\left(d^k_{ij}, d^l_{ij}\right)} \tag{2}$$

n: number of matrix elements that are non-zero in D^k and D^l (Supplementary equation (3))

$$n = \sum_{d^k_{ij} \neq 0 \,\wedge\, d^l_{ij} \neq 0} 1 \tag{3}$$

The correlation for the opposite transposition state $\mathrm{corr}\left(D^k, D^{l^{\mathrm{T}}}\right)$ is calculated accordingly with transposed residue indices of d^l_{ji}.

The resulting correlation c_{kl} is determined as the maximum correlation of both transposition states as shown in Supplementary Equation (4).

$$c_{kl} = \max\left[\mathrm{corr}(D^k, D^l), \mathrm{corr}\left(D^k, D^{l^{\mathrm{T}}}\right)\right] \tag{4}$$

Parameter for Partitioning using the Louvain Method

Louvain partitioning [36] is performed using the implementation in the *python-louvain* library. The edge weights A_{kl} are set proportional to the correlation coefficients c_{kl} using a proportionality constant of α=1 (see Supplementary Equation (5)).

$$A_{kl} = \alpha c_{kl} \tag{5}$$

Supplementary Method 5: <u>Symmetry Complex Scoring</u>

The clustered symmetry complexes with the highest ipTM+pTM score can, but do not need to be FL predictions. The difference in the model confidence score originates mainly from the difference in the ipTM score due to its predominant proportion of 80%. The ipTM score is an accuracy estimate calculated from the errors in position between inter-chain residues. A prediction resulting in a relatively rigid assembly and clearly defined domain arrangement is equivalent to small inter-chain residue errors, leading to a high ipTM score. In contrast, domains with a flexible position relative to the assembly are characterized by larger inter-chain residue errors, leading to a low ipTM score. Exemplified by the predictions of *A. salmonicida* the comparison is shown in Figure 2. Predictions with smaller subchains only containing the domains relevant for symmetric assembly give higher ipTM+pTM scores than the FL predictions.

Supplementary Method 6: <u>ipTM+pTM of predicted symmetry complexes</u>

The median ipTM+pTM score of all 18 benchmark cases is presented in this study. Axis A was slightly better predicted than Axis B with a median value of 0.80 and 0.74 respectively. Overall, the median of axis A+B is 0.77.

Supplementary Method 7: <u>Additional steps in *p1* SLPs</u>

To predict low symmetry assemblies the standard workflow of SymProFold was augmented by additional subchains and heterodimer predictions. For each domain, a subchain was created and those were exhaustively predicted as heterodimers with the FL protein. Figure S23A shows the FL model of EA1, an SLP from *B. anthracis,* with the single domain predictions with the highest ipTM+pTM scores. The ipTM+pTM scores of the predictions are shown in Figure S24.

Same steps are shown in the workflow of Figure 1: “Prefiltering”, “Subchain identification”, “Prediction of multimer sets”, “Scoring of symmetry axis complexes”, “Superposition and optimization”, “Parameter extraction”, “Assembly of Unit Cell”, but some of the steps are extended to cover also the situation of a *p1* lattice. The prefiltering step remains unchanged. The standard set of subchains is extended by subchains covering 1 single domain each. In the prediction step, dimer predictions with FL and each single domain are performed. Example results for the dimer predictions of *Bacillus anthracis* (P94217) are shown in a scoring diagram depicted in Figure S24. In the superposition step the layer is built by superposition and the quality score is calculated (see equation (3): $score_{quality} = score_{clash} + score_{bend}$), and unit cell parameters are extracted.

Supplementary Method 8: <u>Crystallographic validation of predicted interfaces</u>

Predicted multimeric domains were expressed heterologous in *E. coli*, purified, and crystallized. The predicted interfaces are reflected in the crystal contacts. Data processing and refinement statistics of the two solved structures are shown in Supplementary Table 6. Statistics generated with phenix.table_one.

Supplementary References

1. Pettersen, E. F. *et al.* UCSF ChimeraX: Structure visualization for researchers, educators, and developers. *Protein Science* **30**, 70–82 (2021).
2. Sogues, A. *et al.* Structure and function of the EA1 surface layer of Bacillus anthracis. *Nat Commun* **14**, 7051 (2023).
3. von Kügelgen, A. *et al.* Membraneless channels sieve cations in ammonia-oxidizing marine archaea. *Nature* **630**, 230–236 (2024).
4. Arbing, M. A. *et al.* Structure of the surface layer of the methanogenic archaean Methanosarcina acetivorans. *Proc Natl Acad Sci U S A* **109**, 11812–11817 (2012).
5. Fujimoto, Z., Hong, L. T. T., Kishine, N., Suzuki, N. & Kimura, K. Tetramer formation of *Bacillus subtilis* YabJ protein that belongs to YjgF/YER057c/UK114 family. *Biosci Biotechnol Biochem* **85**, 297–306 (2021).
6. Streltsov, V. A., Schmidt, P. M. & McKimm-Breschkin, J. L. Structure of an Influenza A virus N9 neuraminidase with a tetrabrachion-domain stalk. *Acta Crystallogr F Struct Biol Commun* **75**, 89–97 (2019).
7. Sagmeister, T. *et al.* The molecular architecture of *Lactobacillus* S-layer: Assembly and attachment to teichoic acids. *Proceedings of the National Academy of Sciences* **121**, (2024).
8. Baranova, E. *et al.* SbsB structure and lattice reconstruction unveil Ca2+ triggered S-layer assembly. *Nature* **487**, 119–122 (2012).
9. Pavkov, T. *et al.* The Structure and Binding Behavior of the Bacterial Cell Surface Layer Protein SbsC. *Structure* **16**, 1226–1237 (2008).
10. Dordic, A. *et al.* Crystallization of domains involved in self-assembly of the S-layer protein SbsC. *Acta Crystallogr Sect F Struct Biol Cryst Commun* **68**, 1511–1514 (2012).
11. Pavkov, T. *et al.* Crystallization and preliminary structure determination of the C-terminal truncated domain of the S-layer protein SbsC. *Acta Crystallogr D Biol Crystallogr* **59**, 1466–8 (2003).
12. Kroutil, M. *et al.* Towards the structure of the C-terminal part of the S-layer protein SbsC. *Acta Crystallogr Sect F Struct Biol Cryst Commun* **65**, 1042–1047 (2009).
13. Kern, J. *et al.* Structure of Surface Layer Homology (SLH) Domains from Bacillus anthracis Surface Array Protein. *Journal of Biological Chemistry* **286**, 26042–26049 (2011).
14. Fioravanti, A. *et al.* Structure of S-layer protein Sap reveals a mechanism for therapeutic intervention in anthrax. *Nat Microbiol* **4**, 1805–1814 (2019).

15. Sychantha, D. *et al.* Molecular Basis for the Attachment of S-Layer Proteins to the Cell Wall of Bacillus anthracis. *Biochemistry* **57**, 1949–1953 (2018).
16. Lanzoni-Mangutchi, P. *et al.* Structure and assembly of the S-layer in C. difficile. *Nature Communications 2022 13:1* **13**, 1–13 (2022).
17. Fagan, R. P. *et al.* Structural insights into the molecular organization of the S-layer from Clostridium difficile. *Mol. Microbiol.* **71**, 1308–1322 (2009).
18. Farci, D. *et al.* The cryo-EM structure of the S-layer deinoxanthin-binding complex of Deinococcus radiodurans informs properties of its environmental interactions. *Journal of Biological Chemistry* **298**, 102031 (2022).
19. von Kügelgen, A., van Dorst, S., Alva, V. & Bharat, T. A. M. A multidomain connector links the outer membrane and cell wall in phylogenetically deep-branching bacteria. *Proc Natl Acad Sci U S A* **119**, e2203156119 (2022).
20. von Kügelgen, A. *et al.* Interdigitated immunoglobulin arrays form the hyperstable surface layer of the extremophilic bacterium Deinococcus radiodurans. *Proc Natl Acad Sci U S A* **120**, e2215808120 (2023).
21. Herdman, M. *et al.* High-resolution mapping of metal ions reveals principles of surface layer assembly in Caulobacter crescentus cells. *Structure* **30**, 215-228.e5 (2022).
22. von Kügelgen, A. *et al.* In Situ Structure of an Intact Lipopolysaccharide-Bound Bacterial Surface Layer. *Cell* **180**, 348-358.e15 (2020).
23. Herrmann, J. *et al.* A bacterial surface layer protein exploits multistep crystallization for rapid self-assembly. *Proc Natl Acad Sci U S A* **117**, 388–394 (2020).
24. Bharat, T. A. M. *et al.* Structure of the hexagonal surface layer on Caulobacter crescentus cells. *Nat Microbiol* **2**, 17059 (2017).
25. von Kügelgen, A., Alva, V. & Bharat, T. A. M. Complete atomic structure of a native archaeal cell surface. *Cell Rep* **37**, 110052 (2021).
26. Jing, H., Takagi, J., Liu, J. & Springer, T. A. Archaeal Surface Layer Proteins Contain β Propeller, PKD, and β Helix Domains and Are Related to Metazoan Cell Surface Proteins. *Structure* **10**, 1453–1464 (2002).
27. Gambelli, L. *et al.* Structure of the two-component S-layer of the archaeon Sulfolobus acidocaldarius. *bioRxiv* (2022) doi:10.1101/2022.10.07.511299.
28. Gambelli, L. *et al.* Structure of the two-component S-layer of the archaeon Sulfolobus acidocaldarius. *Elife* **13**, (2024).
29. Steinegger, M. & Söding, J. MMseqs2 enables sensitive protein sequence searching for the analysis of massive data sets. *Nat Biotechnol* **35**, 1026–1028 (2017).

30. Zimmermann, L. *et al.* A Completely Reimplemented MPI Bioinformatics Toolkit with a New HHpred Server at its Core. *J Mol Biol* **430**, 2237–2243 (2018).
31. Tickle, I. *et al.* The STARANISO Server. *https://staraniso.globalphasing.org/cgi-bin/staraniso.cgi* (2024).
32. Wu, T., Hou, J., Adhikari, B. & Cheng, J. Analysis of several key factors influencing deep learning-based inter-residue contact prediction. *Bioinformatics* **36**, 1091–1098 (2020).
33. Haghani, M. NEFFy: NEFF Calculator and MSA File Converter. *https://github.com/Maryam-Haghani/Neffy* (2024).
34. Varadi, M. *et al.* AlphaFold Protein Structure Database: Massively expanding the structural coverage of protein-sequence space with high-accuracy models. *Nucleic Acids Res* **50**, D439–D444 (2022).
35. Hiranuma, N. *et al.* Improved protein structure refinement guided by deep learning based accuracy estimation. *Nat Commun* **12**, 1340 (2021).
36. Blondel, V. D., Guillaume, J.-L., Lambiotte, R. & Lefebvre, E. Fast unfolding of communities in large networks. (2008) doi:10.1088/1742-5468/2008/10/P10008.
